# From biomechanical mechanisms to clinical reasoning: Deciphering mechanobiological drivers of aortic valve calcification for precision therapy

**DOI:** 10.1016/j.mbm.2026.100208

**Published:** 2026-08-22

**Authors:** Yefan Jiang, Yuqiu He, Qiong Jia, Si Chen, Youhua Tan, Yongfeng Shao

**Affiliations:** a Department of Cardiovascular Surgery, The First Affiliated Hospital of Nanjing Medical University, Nanjing, 210000, China; b The Hong Kong Polytechnic University Shenzhen Research Institute, Shenzhen, 518057, China; c Research Institute of Smart Ageing, The Hong Kong Polytechnic University, Hong Kong, 999077, China; d Department of Biomedical Engineering, The Hong Kong Polytechnic University, Hong Kong, 999077, China; e Department of Oncology, Nanjing First Hospital, Nanjing Medical University, Nanjing, 210000, China; f Department of Cardiovascular Surgery, Wuhan Union Hospital, Huazhong University of Science and Technology, Wuhan, 430000, China; g Department of Cardiovascular Surgery, The Friendship Hospital of Ili Kazakh Autonomous Prefecture, Ili and Jiangsu Joint Institute of Health, Ili, 835000, China

**Keywords:** Mechanobiology, Calcific aortic valve disease, Traditional chemical signaling pathways, Mechanical force signaling pathways, Mechanomedicine

## Abstract

Calcific aortic valve disease (CAVD) is a degenerative cardiovascular disorder characterized by progressive valvular thickening and calcification, ultimately leading to aortic stenosis, heart failure, and high mortality. Despite associations with hyperlipidemia, inflammation, and bicuspid aortic valve (BAV) malformation, no targeted pharmacotherapies exist, indicating unaddressed core pathogenic mechanisms. The aortic valve is composed of valvular interstitial cells (VICs) and endothelial cells (VECs) which are constantly exposed to hemodynamic forces, with progressive extracellular matrix (ECM) stiffening during CAVD—two mechanobiological pillars closely linked to pathogenesis. This article synthesizes current mechanobiology research on CAVD and bridges biomechanical insights with clinical practice to dissect precision interventions. Clinical and experimental evidence confirms abnormal hemodynamics (e.g., oscillatory shear stress, elevated blood pressure) and increased ECM stiffness drive CAVD by modulating VIC phenotypic transition (osteogenic differentiation, myofibroblastic transformation), VEC dysfunction (epithelial-mesenchymal transition, inflammation), and ECM remodeling/apoptosis. These effects are mediated by chemical pathways (Transforming growth factor-β1(TGF-β1), YAP, Notch1/Runt-related transcription factor 2 (Runx2), Wnt/β-catenin) and cytoskeleton/nucleoskeleton-dependent mechanotransduction (integrin-linker of the nucleoskeleton and cytoskeleton (LINC) complex-chromatin cascade). Current research uses bioreactors and stiffness-tunable gels but faces challenges of unstandardized parameters, decoupled mechanical cues, and limited clinical specimens. Derived insights enable mechanomedicine innovations: novel drug targets, physical therapies (ultrasound/light-mediated ECM modulation), personalized surgical planning (computational fluid dynamics-guided valvuloplasty), optimized bioprosthetic valves, and early diagnosis (valvular elastography). Overcoming interdisciplinary barriers and standardizing methods will accelerate translation of mechanobiological findings into precision clinical strategies, addressing CAVD patients’ unmet needs.

## Graphical abstract

To explore the role of mechanobiology in the progression of calcific aortic valve disease from both clinical and basic perspectives.

**Figure:**
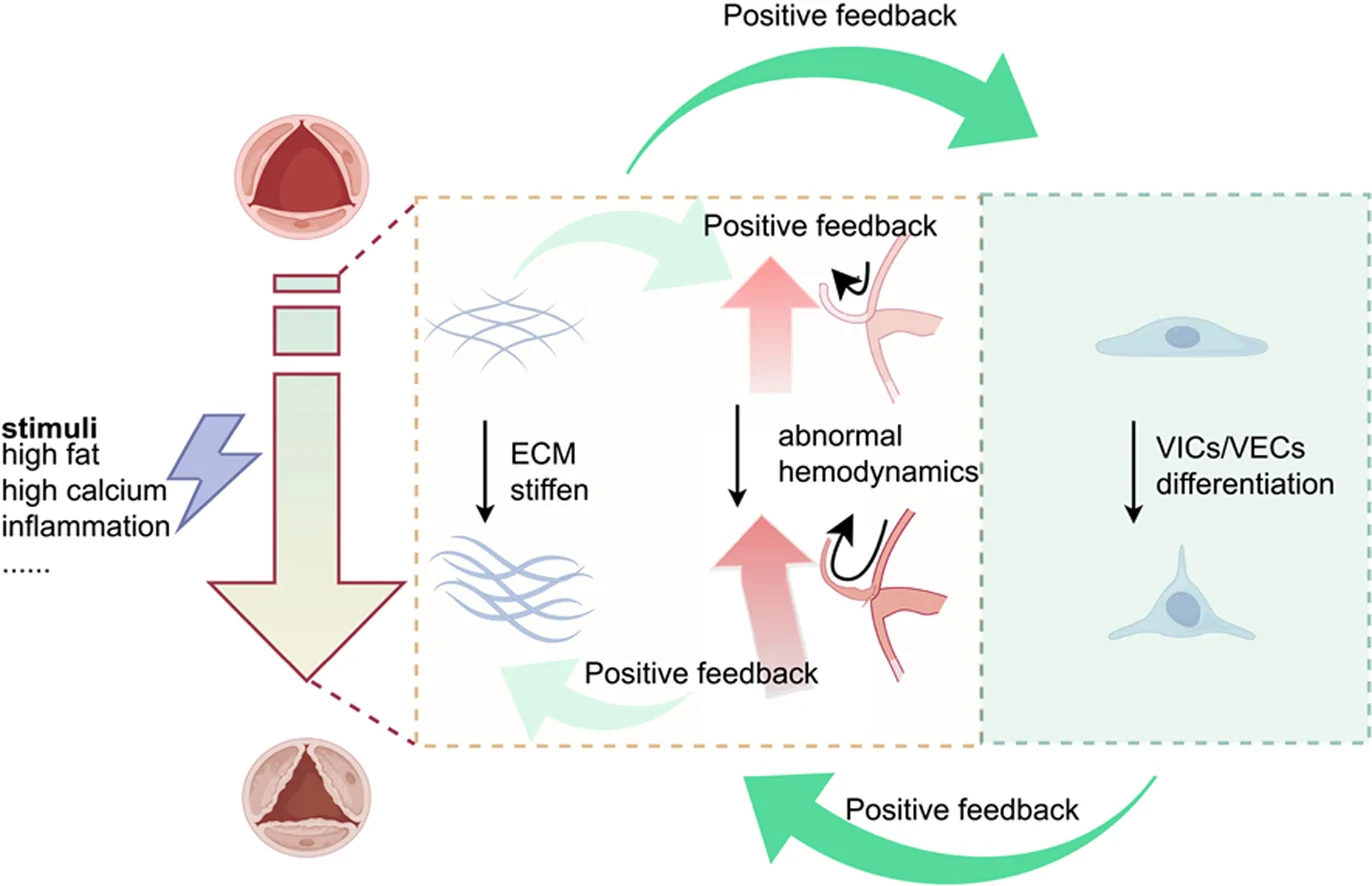

## 1 Introduction

Calcific aortic valve disease (CAVD) is a degenerative disease characterized primarily by the thickening and calcification of the aortic valves, potentially leading to aortic stenosis, heart failure, and even life-threatening outcomes.^1^^,^^2^ Patients with moderate to severe aortic stenosis have a five-year mortality rate of 56% and 67%, respectively.^3^ Currently, there are no pharmacological treatments available for CAVD, with surgical interventions such as thoracotomic or transcatheter aortic valve replacement being the main therapeutic approaches.^4^ (see Table 1).

**Table 1:** | Pharmacotherapeutic targets and clinical evidence for calcific aortic valve disease, stratified by pathogenic initiating drivers.

Initiating Driver	Therapeutic Target	Pharmacologic Agent	Clinical Effect & Evidence
Dyslipidaemia	LDL-C lowering	Statins (rosuvastatin, atorvastatin, simvastatin + ezetimibe)	No benefit on CAVD progression; negative across 4 landmark Randomized controlled trials (RCTs).^5^, ^6^, ^7^, ^8^
	Proprotein convertase subtilisin/kexin type 9 (PCSK9)	PCSK9 inhibitors	Ongoing RCT evaluating valvular calcification and haemodynamic endpoints^9^
	LPA promoter	Niacin	Modestly lowers Lipoprotein(a) (Lp(a)); EAVaLL trial status unknown, limited efficacy expected.^10^
Hypertension	Angiotensin II type 1 receptor	Angiotensin II receptor blockers (ARB)	ALFA RCT ongoing; observational data suggest reduced fibrosis and slower progression.^11^, ^12^, ^13^, ^14^
	Angiotensin-converting enzyme (ACE)	ACE inhibitors	Conflicting, largely neutral results; no consistent haemodynamic benefit.^14^, ^15^, ^16^
Endothelial dysfunction	Soluble guanylate cyclase (sGC)	Ataciguat	CAVS trial completed, results pending publication.^17^
	Dipeptidyl peptidase-4	Evogliptin	No benefit on CAVD progression.^18^
Calcium-phosphate dysregulation	Matrix Gla protein	Vitamin K2	No benefit on CAVD progression.^18^, ^19^, ^20^, ^21^, ^22^
	Hydroxyapatite crystal nucleation and growth	SNF472	Positive phase 2b; ∼86% inhibition of calcification progression in haemodialysis patients.^23^
	RANK ligand	Denosumab	Negative SALTIRE II RCT; no effect on calcification or haemodynamic progression.^24^
	Osteoclast-mediated bone resorption	Alendronic acid	Negative SALTIRE II RCT; no valvular benefit despite systemic bone turnover suppression.^24^

**Fig. 1:**
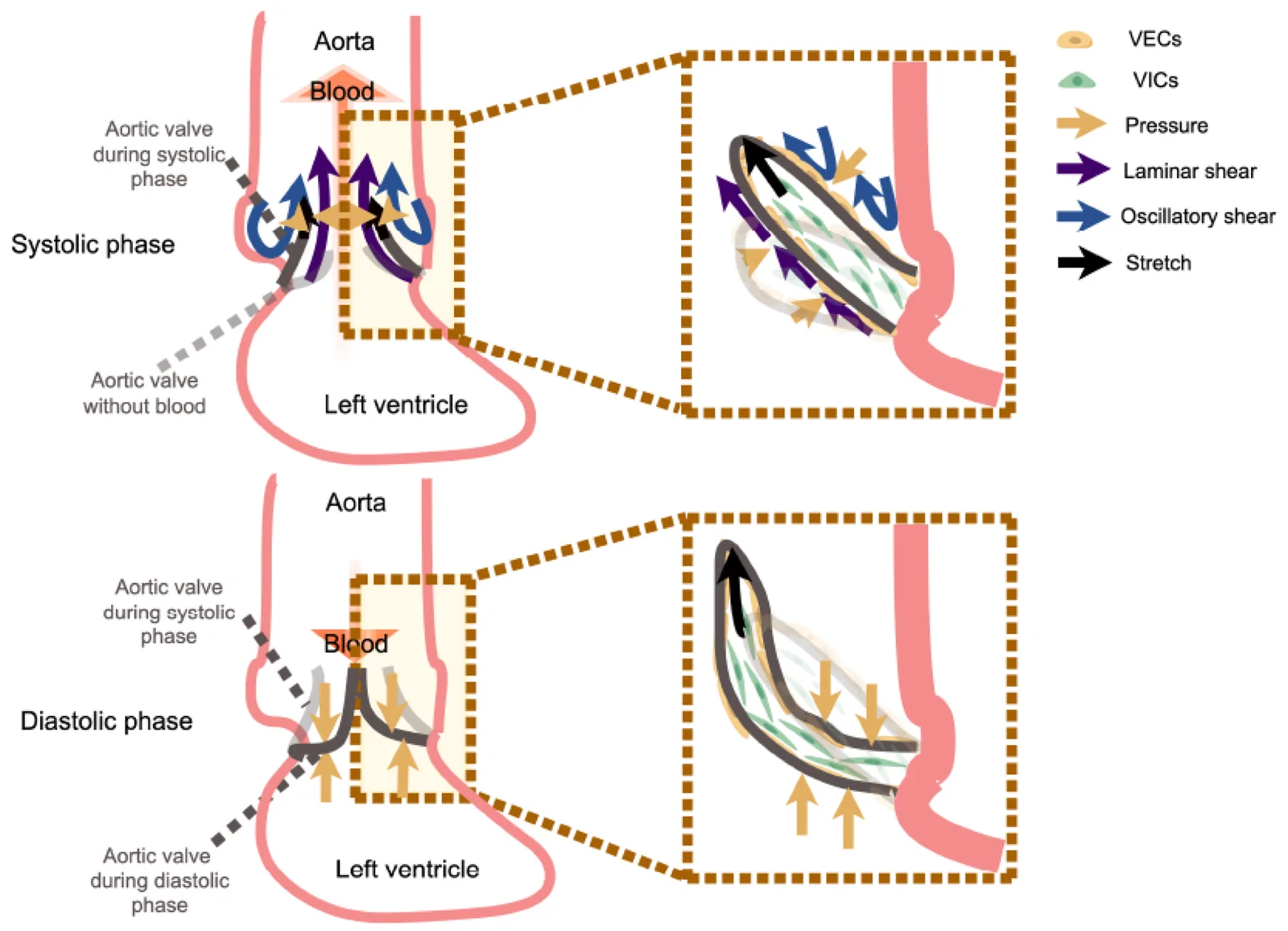
Hemodynamic forces experienced by aortic valves during systolic and diastolic phases This schematic illustrates the dynamic mechanical microenvironment of native aortic valve leaflets during systole and diastole. During systole, the aortic valve opens and blood is ejected from the left ventricle into the aorta; the leaflets are simultaneously subjected to four major mechanical stimuli: transvalvular pressure, laminar shear stress, oscillatory shear stress, and cyclic circumferential stretch. During diastole, the aortic valve closes to prevent blood regurgitation, and the closed leaflets primarily bear sustained transvalvular pressure and tensile stress. The magnified inset depicts the spatial distribution of VECs lining the leaflet surface and VICs embedded within the leaflet matrix, and indicates the direction and distribution pattern of various mechanical loads acting on different cellular layers. This figure delineates the physiological mechanical microenvironment of the aortic valve throughout the cardiac cycle, providing a physiological biomechanical baseline for investigating the mechanobiological mechanisms underlying calcific aortic valve disease (CAVD). Schematic created with FIGDRAW.

The aortic valve, positioned between the left ventricle and the aorta, is composed of VICs and VECs; the phenotypic differentiation of these two cell types constitutes the pathogenic cornerstone of CAVD. It opens and closes passively in response to blood flow, governing unidirectional blood circulation. Statistically, the aortic valves undergo approximately three billion cycles of opening and closing throughout our lifespan, continuously subjected to hemodynamic forces during these processes. During systole, the ventricular side of the aortic valves experiences laminar shear stress and pressure from the blood, while the aortic side experiences oscillatory shear stress and pressure. The valves themselves also undergo stretching due to the forces exerted by the blood. During diastole, the aortic side faces pressure from the blood within the aorta, and the ventricular side is pressured by the blood from the left ventricle, causing the valve leaflet to stretch 46, 47, 48, 49. In CAVD, changes in shear stress, pressure, and stretching forces experienced by the aortic valves underscore the crucial role of hemodynamics in the development and progression of CAVD (Fig. 1). Hemodynamics is one domain of mechanobiology. Another mechanobiological aspect evident in the development and progression of CAVD is valvular stiffness. As CAVD develops, the aortic valves transform from a pliable and thin state to a thick and rigid state. This transition can also modulate the phenotypic alterations of VICs through the stiffness of the extracellular matrix (ECM), subsequently influencing the onset and progression of CAVD.^42^ This prompts the hypothesis that abnormal mechanical microenvironment—including chronic hemodynamic stimuli and altered extracellular matrix (ECM) stiffness—may serve as a core common mechanism mediating multi-etiological CAVD progression. Notably, the causal role of mechanical cues may differ fundamentally across etiological subgroups, acting either as primary initiating drivers or secondary disease amplifiers—a distinction that has not been systematically delineated in prior reviews and has not been sufficiently leveraged for therapeutic development.

## 2 Mechanobiology in relation to the progression of CAVD

### 2.1 Hemodynamic perspectives on CAVD progression

Clinical evidence has established that abnormal hemodynamics play a crucial role in the initiation of aortic valve calcification. In terms of the affected population, patients with BAV exhibit a markedly elevated risk of developing CAVD and an earlier age of disease onset, a phenomenon primarily attributed to the abnormal hemodynamic conditions associated with BAV morphology 50, 51, 52; in terms of the gross morphological characteristics of the valve, valvular calcification predominantly localizes to the valve closure surfaces or extends inwardly from the valvular attachment sites—anatomical regions subjected to maximal bending stress during the systolic and diastolic phases of cardiac valve movement^53^^,^^54^; and in terms of genetic and molecular signatures of the valve, further analyses of surgical specimens have revealed marked dysregulation of cellular pathways responding to fluid shear stress in calcified valves.^55^

#### 2.1.1 The impact of blood pressure on CAVD

During the opening and closing of the aortic valves, both the aortic side and the ventricular side are exposed to pressure from the blood within the aorta and the left ventricle, respectively. Epidemiological data have demonstrated that patients with hypertension have a higher incidence and greater severity of CAVD, with an odds ratio (OR) of 1.23 for CAVD occurrence in hypertensive patients compared with non-hypertensive counterparts.^56^^,^^57^

Blood pressure can influence the development of CAVD through various mechanisms. (1) Regulation of ECM composition: Physiological blood pressure preserves the physiological concentrations of sulfated glycosaminoglycans (sGAGs) and elastin in the aortic valve, thereby safeguarding its structural integrity and biomechanical function. In contrast, elevated blood pressure triggers the straightening and rearrangement of collagen fibers within the valve leaflets, which subsequently contributes to the thinning of the fibrosa and spongiosa layers^58^, ^59^, ^60^. (2) Regulation of fiber distribution in ECM: pressures the fiber-preferred directions coursed along the circumferential direction.^61^ (3) Regulation of inflammatory response: Elevated blood pressure upregulates the expression of a spectrum of pro-inflammatory mediators, including tumor necrosis factor-α (TNF-α), interleukin-1α (IL-1α), and interleukin-1β (IL-1β) in valvular interstitial cells.^35^ (4) Regulation of cell apoptosis: Sustained elevation of blood pressure induces a marked increase in the apoptotic rate of valvular interstitial cells.^59^

#### 2.1.2 Impact of blood shear stress on CAVD

During the opening of the aortic valves, blood passes through the aortic valves, exerting laminar shear stress on the aortic valves' ventricular side. The blood flow reflects off the aortic wall, creating turbulence in the aortic sinuses and inducing vortex-induced oscillatory shear stress on the aortic valve's aortic side.^47^^,^^62^ Calcification predominantly occurs on the aortic sides of aortic valves, a pathological phenomenon correlated with the oscillatory shear stress exerted on this specific anatomical region.^63^ For the aforementioned BAV malformation, laminar shear stress on the fused valvular base correlates positively with aortic valve calcification severity, with this shear stress being 16-fold higher in severely calcified BAVs compared to tricuspid non-calcified valves ^36^^,^^64^^,^^65^.

Blood shear stress can influence the progression of CAVD through multiple mechanisms: (1) Regulation of VICs’ proliferation and phenotype: Laminar shear stress promotes VIC proliferation and myofibroblastic differentiation^66^; (2) Regulation of VEC alignment and differentiation: Laminar shear stress induces VECs to align along the direction of blood flow and maintains their endothelial cell morphology, whereas oscillatory shear stress promotes epithelial-mesenchymal transition (EMT) in VECs 67, 68, 69; (3) Regulation of ECM composition: It modulates the expression of TIMP and MMP to mediate ECM remodeling; laminar shear stress leads to reduced sGAG content, while turbulent shear stress causes thickening of the valvular fibrous layer and increased collagen maturity^51^^,^^63^^,^^66^; (4) Regulation of inflammatory responses: Increased expression of inflammation-related genes such as VCAM1, ICAM1, and NFKB1 has been observed in VECs treated with oscillatory shear stresses.^70^

#### 2.1.3 Impact of blood stretching forces on CAVD

Aortic valves undergo stretching due to laminar shear stresses during opening and experience stretching due to differential blood pressures across the valves during closure. The stretching can specifically be classified into circumferential and radial ones, where circumferential one refers to a stretching parallel to the aortic valve annulus, and a radial one refers to a stretching perpendicular to the aortic valve annulus.^71^ The leaflet area in the open valve state increases by 20% relative to its ex vivo static dimensions, with a further 25% expansion occurring during the transition from the open to the closed state. Such dimensional enlargement promotes the formation of calcific nodules.^72^

Blood stretching forces may influence the progression of CAVD through multiple mechanisms: (1) Regulation of VICs' phenotype, proliferation and apoptosis: A 15% deformation induces myofibroblastic differentiation in VICs, whereas a 20% deformation leads to significant increases in both cell proliferation and apoptosis^24^^,^^43^^,^^72^, ^73^, ^74^, ^75^, ^76^; (2) Regulation of VEC proliferation and apoptosis: A 15% deformation induced by stretching forces significantly enhances the viability of VECs^77^; (3) Regulation of ECM composition: Deformations of varying magnitudes modulate the expression of matrix metalloproteinases (MMPs) and tissue inhibitors of metalloproteinases (TIMPs), thereby altering collagen content and maturity^43^^,^^74^^,^^76^^,^^78^; (4) Regulation of inflammatory responses: Deformations could down-regulate the expression of pro-inflammatory factors.^37^

### 2.2 ECM stiffness perspectives on CAVD progression

Aortic valve stiffening represents a core pathological change in CAVD. Whether induced by abnormal hemodynamics or other pathogenic factors, the phenotypic transformation of VICs is always accompanied by VIC-mediated ECM remodeling, which is specifically characterized by increased contents of collagen, proteoglycans, and glycosaminoglycans, coupled with decreased elastin levels.^42^ Despite changes in ECM composition that modulate CAVD progression, alterations in ECM stiffness also exert a regulatory effect on CAVD development.^23^ While specific stiffness values for normal versus calcified aortic valves have not been fully defined, valve stiffening during CAVD progression is a well-established consensus, and in vitro stiffness models developed based on this phenomenon have confirmed the regulatory role of ECM stiffness in VIC differentiation.^79^^,^^80^

#### 2.2.1 Impact of ECM stiffness on CAVD

Extracellular matrix (ECM) stiffness exerts a multifaceted regulatory effect on the progression of calcific aortic valve disease (CAVD). The specific mechanisms are elaborated as follows: (1) Regulation of VICs phenotype, proliferation, and apoptosis: A softer ECM microenvironment is capable of maintaining VICs in a quiescent state, which is highly consistent with the phenotypic characteristics of freshly isolated primary VICs.^25^^,^^81^ With the gradual increase in ECM stiffness, VICs undergo a sequential phenotypic transition: initially differentiating toward the osteogenic lineage, and further elevation of ECM stiffness drives a shift in their differentiation trajectory from osteogenic differentiation to myofibroblastic transformation.^82^ Importantly, the regulatory effect of ECM stiffness on VICs phenotype is reversible.^25^^,^^83^^,^^84^ In addition, ECM stiffness also plays a pivotal role in modulating the proliferation and differentiation of VICs; Furthermore, the changes in VICs' phenotypes are also related to the uniformity and surface structure of the ECM.^81^^,^^84^ (2) Regulation of VECs phenotypic transformation: Distinct ECM stiffness levels induce divergent phenotypic differentiation patterns in VECs. Specifically, a stiffer ECM tends to promote the EMT process of VECs, whereas a softer ECM is conducive to maintaining the viability and endothelial phenotypic stability of VECs^29^^,^^85^^,^^86^. (3) Regulation of ECM composition: A stiffer ECM can downregulate the activity of ECM-degrading enzymes, such as tissue inhibitor of metalloproteinases 3 (TIMP3), thereby affecting the dynamic remodeling balance of the ECM.^25^

In summary, hemodynamics (blood pressure, shear stress, and tensile stress) and ECM stiffness drive CAVD progression through conserved multidimensional cellular mechanisms. Critically, the pathogenetic role of these mechanobiological factors is not uniform across patient subgroups, but follows a clear dichotomous framework of initiating drivers vs. secondary amplifiers—a core conceptual distinction elaborated in this review.

In congenital etiology subgroups, mechanobiological abnormalities act as primary initiating drivers of disease onset: in BAV, aberrant hemodynamic shear stress and elevated cyclic strain precede valvular calcification and directly trigger pathological cellular transition; in Marfan syndrome, inherent ECM structural defects and abnormal matrix stiffness represent the primary pathogenic insult that initiates CAVD.^87^

**Fig. 2:**
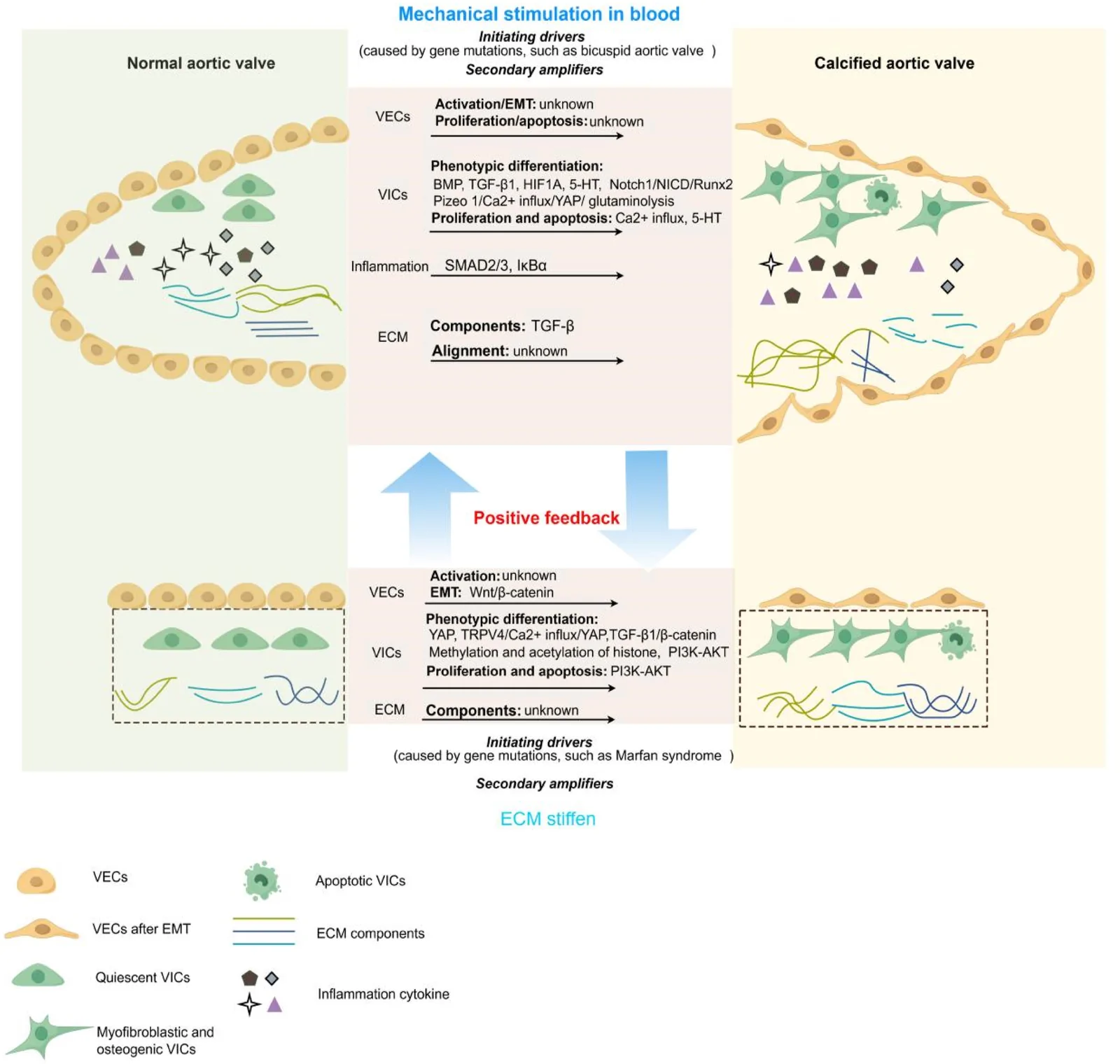
Traditional chemical signaling pathways involved in mechanobiology affecting CAVD This figure systematically presents two types of initiating mechanical injuries and their downstream chemical signaling cascades that drive the progression of calcific aortic valve disease (CAVD), which are interconnected and mutually reinforced via a positive feedback loop. Hemodynamic abnormalities can be either triggered by initiating factors such as bicuspid aortic valve malformation and hypertension, or occur secondarily during disease progression. Increased extracellular matrix (ECM) stiffness can likewise represent an initiating change mediated by etiologies such as Marfan syndrome, or arise secondary to disease progression. Abnormal blood flow promotes increased ECM stiffness, which in turn exacerbates hemodynamic abnormalities and drives their progression. The two thereby form a self-reinforcing positive feedback loop that collectively accelerates the disease course of CAVD. Disturbed hemodynamic mechanical signals induce the activation of VECs and potential endothelial-to-mesenchymal transition (EMT), accompanied by an imbalance between cell proliferation and apoptosis. In VICs, these mechanical signals mediate cellular differentiation toward myofibroblast-like and osteoblast-like phenotypes through multiple pathways including BMP, TGF-β1, HIF1A, YAP, and Piezo1/calcium influx. Meanwhile, inflammatory signals mediated by SMAD2/3 and IκBα further amplify the damaging effects, ultimately resulting in abnormal remodeling and disorganized fiber arrangement of ECM components. Elevated matrix stiffness, in turn, induces VEC activation and EMT via the Wnt/β-catenin pathway, drives VIC phenotypic transition through YAP, TRPV4/calcium influx, PI3K-AKT, and epigenetic modification pathways, and further aggravates ECM dysregulation and structural remodeling. Schematic created with FIGDRAW.

In contrast, in the vast majority of acquired CAVD cases driven by classical risk factors such as hyperlipidemia and chronic inflammation, mechanobiological alterations function as secondary amplifiers rather than initial triggers. Extrinsic pathogenic stimuli first induce valvular thickening and sclerosis; these structural changes in turn distort valvular hemodynamics and increase local matrix stiffness, which further promotes valvular cellular phenotypic transition and pathological matrix remodeling, forming a self-sustaining positive feedback loop that accelerates disease progression (Fig. 2).

### 2.3 Signaling pathways involved in mechanobiology affecting CAVD

Current studies on the mechanical impacts on CAVD largely focused on phenomena, such as VICs’ phenotypic differentiation or changes in ECM composition under mechanical stimuli, with limited exploration into the signaling pathways involved. Notably, there is a scarcity of studies detailing the specific signal transduction pathways by which blood pressure affects CAVD. The following summarizes available information on related signaling pathways.

#### 2.3.1 Traditional chemical signaling pathways

Stretching forces can impact the progression of CAVD by influencing the osteogenic differentiation of VICs through the Bone morphogenetic protein (BMP) and TGF-β1 signaling pathways.^24^^,^^38^ Moreover, those forces can also regulate ECM composition, thus affecting CAVD progression through the Hypoxia-inducible factor 1A (HIF1A) and 5-HT signaling pathways,^39^^,^^76^ as well as by modulating Ca^2+^ influx to affect VICs' apoptosis.^75^ Additionally, the 5-hydroxytryptamine 2A receptor (5-HT_2A)_ receptor signaling pathway can also alter VICs’ proliferation, thereby influencing CAVD progression.^76^ Furthermore, inflammation related to CAVD can be regulated by modulating the phosphorylation of SMAD 2/3 and IκBα^37^ (Fig. 2).

The study investigating the impact of shear stress on the progression of CAVD by modulating VICs’ osteogenic differentiation provides detailed insights into the signaling pathways involved. Shear stress can activate the Piezo1 channel, leading to Ca^2+^ influx and subsequent nuclear translocation of YAP, which in turn promotes GLS1-mediated glutamine breakdown. This process increases intracellular acetyl-CoA levels and enhances histone H3 acetylation, thereby promoting the osteogenic differentiation of VICs and affecting CAVD progression.^34^ Moreover, Li et al. also suggested that shear stress could regulate VICs' osteogenic differentiation through the Notch1/NICD/Runx2 signaling pathway.^36^ Besides, shear stress can also modify ECM composition through the TGF-β1 signaling pathway, thus affecting CAVD progression^63^ (Fig. 2).

The current understanding of the signaling pathways by which ECM stiffness influences CAVD primarily centers on its impact on VICs' phenotypic differentiation. Signaling pathways reported are listed as follows: (1) promoting nuclear translocation of YAP; (2) regulating the Phosphoinositide 3-kinase/protein kinase B (PI3K-AKT) signaling pathway; (3) modulating the expression of the mechanosensitive calcium channel Transient receptor potential vanilloid 4 (TRPV4), leading to YAP nuclear translocation following Ca^2+^ influx; (4) controlling methylation and acetylation of histones; (5) facilitating nuclear translocation of β-catenin via TGF-β1 25, 26, 27^,^^30^^,^^81^. Further studies indicate that ECM stiffness can regulate VICs’ apoptosis through phosphorylation of AKT or promote the EMT process in VECs through the Wnt/β-catenin pathway, thereby impacting CAVD progression^29^^,^^82^ (Fig. 2).

#### 2.3.2 Mechanotransduction mediated by cytoskeletal proteins

**Fig. 3:**
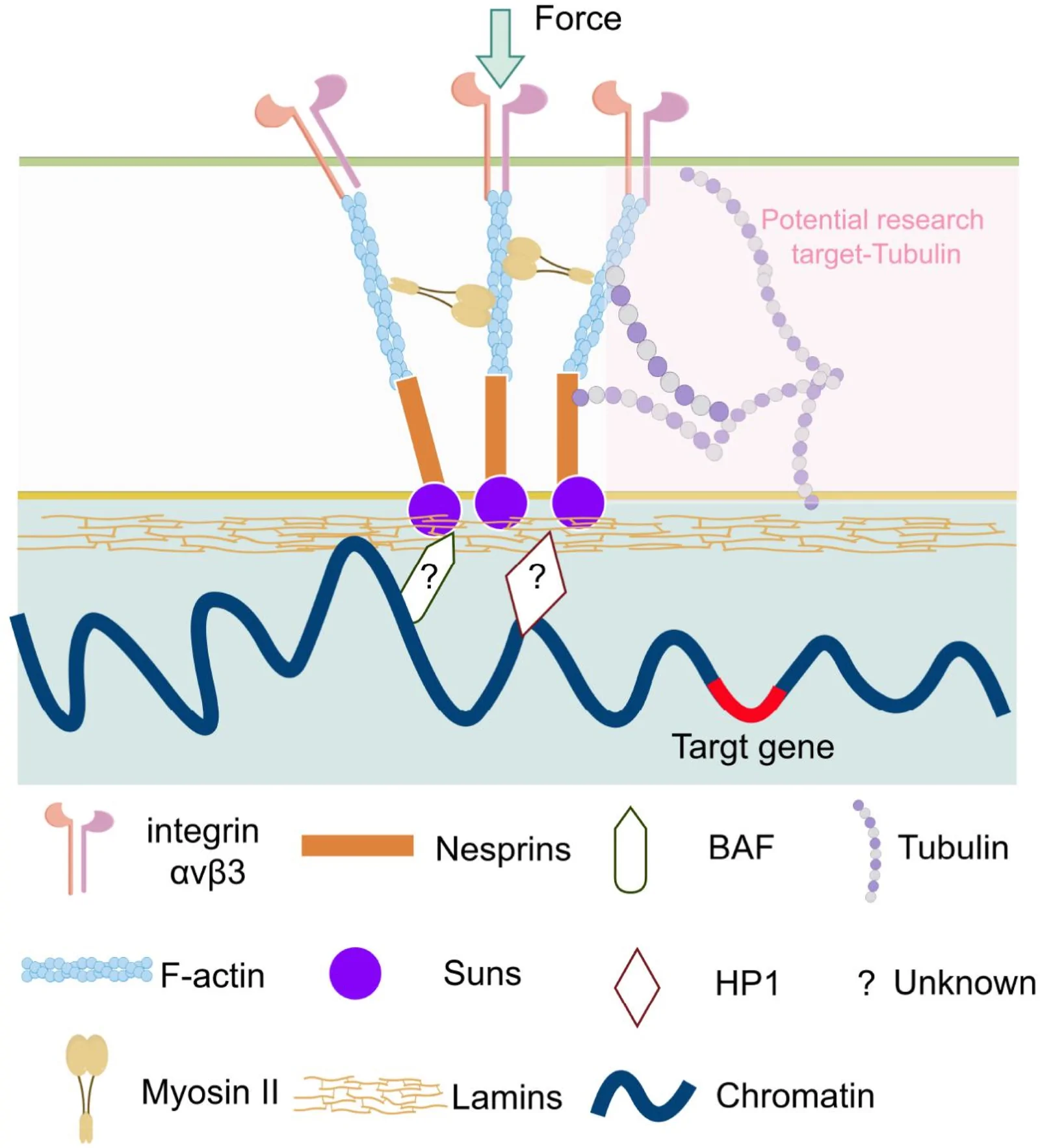
Mechanical force signaling pathways mediated by cytoskeletal proteins involved in mechanobiology affecting CAVD This schematic demonstrates the direct mechanotransduction axis through which extracellular mechanical forces are transmitted from the cell surface to the nucleus to regulate target gene expression in calcific aortic valve disease (CAVD). Extracellular mechanical forces are first sensed by the transmembrane integrin αvβ3 receptor, and then propagated intracellularly via the F-actin cytoskeleton and myosin Ⅱ. The mechanical signals further traverse the nuclear envelope through components of the linker of nucleoskeleton and cytoskeleton (LINC) complex, namely Nesprins and inner nuclear membrane proteins, and are transmitted to lamins and intranuclear SUN proteins. This physical force transmission process remodels the spatial conformation of chromatin and regulates the expression of target genes related to osteogenic differentiation and ECM remodeling via intranuclear mediators such as BAF and HP1. This "cytoskeleton–nucleus" mechanical coupling constitutes a direct mechanotransduction pathway independent of chemical messengers, which converts extracellular biomechanical signals into sustained cellular phenotypic alterations during CAVD progression. Additionally, microtubules, as a core component of the cytoskeleton, stabilize cellular structure through compressive action and participate in mechanical signal transduction by coupling with the nucleus and YAP protein. However, their roles in the mechanobiological regulation of VICs and VECs, as well as in the pathological progression of CAVD, have not been fully elucidated, representing an important future research direction in this field. Schematic created with FIGDRAW.

In addition to the traditional chemical signaling pathways, mechanoskeletal transduction also mediates the regulatory effects of mechanical stimuli on the progression of CAVD^88^, ^89^, ^90^. This regulatory role is reflected not only in altered expression levels of cytoskeletal proteins but also in the rearrangement of these proteins in response to external mechanical stimuli^88^, ^89^, ^90^. Such rearrangement modulates cellular morphology to resist external mechanical stresses, accompanied by corresponding changes in cellular properties including aspect ratio and intrinsic stiffness ^31^^,^^91^^,^^92^ (Fig. 3).

Mechanoskeletal transduction of mechanical signals denotes a highly ordered, sequential cascade in which extracellular mechanical stimuli being transmitted through mechanoreceptors such as integrins to the cytoskeleton (e.g., actin/myosin II). This stimulus is further conveyed to the nucleoskeleton (e.g., Lamin A/C) via the LINC complex (including Nesprins and Suns). Once in the nucleoskeleton, the stimuli are transmitted to chromatin through BAF and HP1 proteins, which connect the nucleoskeleton to chromatin. Then, chromatin is decondensed, directly regulating the expression of target genes.^28^^,^^32^ Notably, as critical cytoskeletal components, microtubules have been relatively understudied in prior investigations into the biomechanical regulation of CAVD. Serving as core load-bearing and mechanosensitive cytoskeletal elements, they function as compression-stabilized mechanotransducers that couple mechanical cues to the nucleus and YAP signaling^93^, ^94^, ^95^, ^96^, ^97^. In parallel, microtubules interdigitate with actin and myosin filaments, thereby indirectly modulating the mechanoregulatory effects of the actomyosin cytoskeleton. Accordingly, tubulin, the core structural subunit of microtubules, has yet to be thoroughly characterized in the mechanical modulation of CAVD and stands as a high-value direction for future research.

Mechanotransduction of cytoskeletal proteins is classified into two types. The first type depends on the dissociation and polymerization of cytoskeletal proteins: upon exposure to external mechanical stimuli, the cytoskeleton undergoes morphological remodeling to counteract the stimuli, and the stress generated by this deformation is transmitted to the nucleoskeleton. In response, the nucleoskeleton undergoes conformational adjustments to mitigate such stress.^31^^,^^91^ During this process, the LINC complex further relays the stress to chromatin. This constitutes a passive transduction mode, whose core function is to resist external mechanical stimuli, with stimulus intensity positively correlating with the degree of cellular deformation. Current research on mechanotransduction in CAVD has primarily focused on this mode. Specifically, under external stimulation, actin in VICs polymerizes into filamentous actin or combines with myosin to form actomyosin complexes.^98^ Notably, this process may also involve the polymerization of α-SMA and vimentin, with α-SMA polymerization regulated by the magnitude of stretching forces and vimentin polymerization governed by the rate of stretching force changes.^40^^,^^99^ Mechanical signals are subsequently transmitted to the nucleus via LINC complex components (e.g., SUN proteins and Nesprins), accompanied by altered distribution of Lamin A/C.

Although previous researches have reported that Lamins can directly link the LINC complex with chromatin, Tajik et al. discovered that in the mechanical force signaling pathways, the transmission of a mechanical signal from the LINC complex to Lamins requires further passage through BAF, HP1, or other proteins to ultimately reach the chromatin 28,32. Current studies on how mechanical signals are transferred from the nucleoskeleton to chromatin in CAVD are still lacking. However, the significant alterations in nuclear circularity and chromatin condensation parameters under mechanical stimulation unequivocally demonstrate the existence of mechanical signal transduction mediated by the nucleoskeleton.^33^^,^^100^

The second type of cytoskeletal mechanotransduction does not rely on cytoskeleton polymerization and depolymerization, but on the oscillation of cytoskeletal proteins.^32^ It achieves signal transduction via reversible conformational oscillations of cytoskeleton-associated proteins containing tandem spectrin repeat (SR) domains. Under physiological piconewton-scale mechanical forces, the tandem SR domains of these proteins undergo stochastic, dynamic unfolding and refolding transitions. The extension and retraction of these domains buffer mechanical stress and stably maintain tension along the cytoskeletal force transmission pathway within a physiological range of 5–10 pN, acting as a molecular shock absorber^93^^,^^101^, ^102^, ^103^, ^104^. Unlike the first signaling pathway, which involves the participation of chemical signaling cascades, the second pathway is purely mediated by cytoskeletal proteins—this unique mechanism enables the compression of relatively long signal transduction timeframes to within a few seconds and does not alter the overall cytoskeletal architecture.^30^^,^^82^^,^^105^ Using a magnetic twisting cytometer (MTC), Jiang et al. provided indirect supporting evidence for the potential existence of this cytoskeleton-only signaling pathway in CAVD, by demonstrating transient gene expression changes in VICs under mechanical stimulation. However, the immediate RNA isolation approach used in their study carries inherent methodological limitations and cannot fully exclude interference from concurrently activated rapid chemical signaling cascades. To definitively confirm this purely mechanical transduction pathway, further validation using real-time imaging of instantaneous intranuclear fluorescence changes—an experimental paradigm pioneered by Professor Ning Wang's group—is still required^32^.^106^

### 2.4 Mechanical research methods for CAVD: advantages and key limitations

Current research on the mechanical influences of CAVD primarily employs bioreactors and gels: bioreactors simulate the blood pressure, stretching forces, and shear stress endured by aortic valves in vivo to study hemodynamic impacts, while gels enable exploration of ECM stiffness effects by adjusting their composition to replicate diverse stiffness levels.

The key merits of these approaches are as follows: first, bioreactors can replicate individual or combined hemodynamic forces (blood pressure, stretching forces, shear stress) that aortic valves endure in vivo, a capability unattainable in animal models—unlike animal models (e.g., gene-modified mice, injured mouse models, or porcine models) that face limitations such as confounding genetic effects, operational complexity, anatomical discrepancies with humans, or high costs, bioreactors enable precise differentiation of the roles of distinct mechanical cues. Second, bioreactors feature adjustable parameters, facilitating exploration of the effects of varying magnitudes and frequencies of hemodynamic forces on CAVD progression. Third, bioreactors support mechanical stimulation of both isolated cells and valve tissues, offering multi-scale research perspectives, while gels can simulate different ECM stiffness levels by adjusting their composition, providing a targeted tool for studying ECM stiffness-related cellular regulation. Fourth, bioreactors allow controlled drug interventions alongside mechanical stimulation to assess pharmaceutical effects on CAVD, and gels are widely adopted due to their simplicity of use, cost-effectiveness, and controllability. Fifth, bioreactors can partially replace animal models, compensating for the ethical and practical constraint of in vivo animal experiments; however, animal models remain indispensable for validating cellular findings due to the lack of inter-organ interactions in bioreactors^34^^,^^107^, ^108^, ^109^, ^110^, ^111^, ^112^, ^113^, ^114^.

Despite their advantages, these research methods still face critical challenges. First, there is a lack of standardized, validated in vivo reference values for hemodynamic parameters and valve stiffness^29^^,^^30^^,^^47^^,^^82^^,^^93^^,^^115^, ^116^, ^117^, ^118^, ^119^, ^120^. Supplementary Tables 1–4 summarize the hemodynamic and stiffness values of aortic valves derived from present measurements, alongside the hemodynamic parameters and stiffness characteristics of normal and CAVD valves used in existing literature, respectively. Taken together, published in vivo data on hemodynamic parameters and valvular stiffness are highly heterogeneous under both normal and diseased conditions; no consensus reference values have been validated, and inter-study discrepancies can be as great as an order of magnitude. Second, it is difficult to decouple interconnected mechanical stimuli present under physiological conditions (e.g., elevated blood pressure amplifies valvular circumferential and radial stretching).^121^ Third, many bioreactors fail to replicate the pulsatile nature of in vivo blood flow, which is critical to research validity^122^^,^^123^; meanwhile, the choice between two-dimensional (2D) and three-dimensional (3D) cultures of VICs significantly impacts conclusions about ECM stiffness regulation in gel-based studies.^124^ Fourth, most reported bioreactors and gel-based experimental setups are custom-developed by individual research groups without rigorous testing and calibration; only a few studies have verified that the flow generated by bioreactors matches in vivo blood flow characteristics, and gel-based stiffness simulations lack systematic validation^51^^,^^62^^,^^125^

### 2.5 Critical barriers to biomechanical investigations in CAVD

In the preceding sections, we have synthesized the current landscape of research elucidating the role of biomechanics in the progression of CAVD. From a clinical perspective, the aortic valves are perpetually subjected to dynamic hemodynamic forces, rendering the impact of mechanical cues on disease pathogenesis indisputable. Nevertheless, research in this domain remains relatively sparse, attributable to the following key constraint.

**Low disease prevalence:** While CAVD constitutes a critical subset of cardiovascular disorders, its incidence is substantially lower than that of highly prevalent conditions including coronary artery disease and systemic hypertension.

**Asymptomatic early-to-moderate stages:** Although end-stage CAVD is closely associated with increased left ventricular dysfunction, elevated mortality, and a life expectancy of approximately 5 years following symptom onset, patients in the early, middle, and even some advanced stages often remain asymptomatic and do not impair daily activities—contributing to infrequent medical consultations and insufficient awareness of the disease's severe sequelae. Additionally, prior to the development of left ventricular enlargement, CAVD is challenging to detect via routine physical examination modalities (e.g., electrocardiography, chest radiography, chest CT); cardiac auscultation requires high professional proficiency, leading to low rates of heart murmur detection among non-specialists, while echocardiography—the primary modality for CAVD diagnosis—is not a routine component of general health screenings.

**Restricted accessibility to clinical specimens:** Cardiac surgeries are highly specialized and technically demanding procedures, thus predominantly concentrated in a limited number of tertiary referral centers. This geographic and institutional concentration results in the restricted accessibility of CAVD-related clinical specimens. In stark contrast to research in oncology or atherosclerosis—where animal models and established cell lines are widely utilized as surrogate research systems—such experimental resources remain notably scarce for CAVD mechanistic investigations.

Hence, in line with research paradigms for other major diseases such as tumors, the establishment of dedicated public databases—which should encompass, but not be limited to, data derived from genetic or proteomic assays of clinical specimens, measurements of biomechanically relevant parameters, as well as resources for the sharing of patient-derived specimens and clinical datasets pertaining to population-level incidence and long-term prognosis—and active encouragement of scholars to share primary research data represent a critical prerequisite for robust mechanobiology-based explorations of CAVD pathogenesis and therapeutic strategies. Additionally, the development of validated murine models that circumvent the limitations of large animal models (including prohibitive costs and prolonged breeding cycles), together with the establishment of stable cell lines and the deployment of gene chip technology, will also provide valuable insights to advance CAVD research 107, 108, 109, 110, 111, 112, 113, 114^,^^126^^,^^127^.

**The niche interdisciplinary nature of biomechanics:** As a cross-disciplinary field integrating principles of biology and mechanical engineering, biomechanical research necessitates specialized instrumentation and technical expertise. The relatively small cadre of researchers with proficiency in both domains further conshear stresss the pace of research progress in this area.

**Challenges in clinician-scientist collaboration, the most critical barrier:** This bottleneck arises from the extremely narrow intersection between the relatively concentrated clinical community specializing in valvular heart disease and the small cohort of biomechanics researchers. Despite a shared consensus among clinicians and biomechanics investigators regarding the pivotal role of mechanical forces in CAVD pathogenesis, identifying mutually compatible collaborators with complementary expertise remains a persistent and formidable challenge.

### 2.6 Mechanomedicine: A promising paradigm for addressing unmet clinical needs in calcific aortic valve disease

While research exploring the utility of mechanomedicine in the management of CAVD from a mechanobiological standpoint is still relatively scarce and confronted with multiple hurdles,the inherent reversibility of mechanical effects on CAVD indicates that the distinctive paradigm of innovatively integrating mechanical principles into clinical practice endows this strategy with substantial prospects for clinical translation.^33^^,^^93^^,^^128^^,^^129^ And the central role of mechanical dysregulation as a shared final common pathway across all etiologies. Unlike conventional etiology-specific interventions, mechanomedicine targets the universal biomechanical vicious cycle that drives disease progression regardless of the initial trigger, thus offering broader patient coverage and aligning well with the unmet need for early, disease-modifying therapy.

**First, mechanomedicine-informed drug development**. Traditional signal pathway-based research has not yet yielded therapeutic drugs applicable for CAVD. Since previous studies lacked a mechanobiological focus, investigating mechanotransduction pathways—a core tenet of mechanomedicine—is expected to uncover novel therapeutic targets that are inaccessible via traditional etiology-focused approaches. Unlike research that separately explores individual pathogenic initiators (e.g., inflammation and hyperlipidemia), mechanomedicine ingeniously circumvents the controversy over whether mechanobiological factors act as initiating or secondary contributors to CAVD pathogenesis, enabling targeted investigation of the valvular thickening and stiffening stages induced by all these initiators.^1^^,^^130^ This approach is more aligned with the clinical demand for pharmacotherapy following early diagnosis and avoids the narrow patient applicability of drugs developed via single-factor strategies. For instance, SNF472 has been shown to inhibit hydroxyapatite crystal formation and effectively delay CAVD progression in clinical trials, yet its application is primarily restricted to dialysis-dependent patients with end-stage renal disease.^44^

In contrast, mechanomedicine-derived drugs targeting mechanotransduction pathways could potentially address this limitation by targeting a universal pathological stage rather than a specific etiological factor.^131^^,^^132^ Disruption of this pathogenic mechanobiological positive feedback loop can be strategically achieved by targeting three core pathological axes: (1) valvular cell differentiation driven by progressive ECM stiffening, (2) valvular cell phenotypic transformation triggered by aberrant hemodynamics secondary to valve sclerosis, and (3) pathological extracellular matrix remodeling mediated by phenotypically altered valvular cells. Experimentally validated candidate intervention targets corresponding to each axis are summarized in the table below (see Table 2).

**Table 2:** | Summary of candidate intervention targets targeting the mechanobiological positive feedback loop in calcific aortic valve disease.

Pathological Axis	Target Category	Key Targets
ECM stiffness → valvular cell differentiation	Cell membrane mechanosensory targets	Integrins, TRPV4 channels^25^, ^26^, ^27^, ^28^
	Core intracellular signal transduction targets	YAP/TAZ, PI3K/AKT, Wnt/β-catenin, TGF-β1/Smad, epigenetic regulators^25^, ^26^, ^27^^,^^29^^,^^30^
	Cytoskeleton-nucleoskeleton mechanotransduction targets	Actin-myosin cytoskeleton, LINC complex, Lamin A/C^28^^,^^31^, ^32^, ^33^
Abnormal hemodynamics → valvular cell differentiation	Cell membrane mechanosensory targets	Piezo1 channels, 5-HT_2_A receptors, integrins^28^^,^^34^
	Core intracellular signal transduction targets	YAP/TAZ, Notch1/Runx2, TGF-β1/Smad, BMP, HIF1A, Nuclear factor kappa-B (NF-κB)^24^^,^^28^^,^^35^, ^36^, ^37^, ^38^, ^39^
	Cytoskeleton-nucleoskeleton mechanotransduction targets	α-SMA, vimentin, actin-myosin complex, LINC complex, Lamin A/C^28^^,^^32^^,^^40^^,^^41^
Pathological cell differentiation → ECM remodeling	Unified intervention targets	MMP/TIMP regulators, anti-calcification agents (e.g. SNF472), light-mediated ECM crosslinking modulation, TIMP3 upregulation^7^^,^^25^^,^^42^, ^43^, ^44^, ^45^

**Second, mechanomedicine-based physical therapeutic strategies for CAVD**. As a non-pharmacological intervention directly targeting the tissue mechanical microenvironment, physical therapy represents one of the most direct translational outputs of mechanobiological research, and can be stratified into matrix-stabilizing and calcification-reversing strategies tailored to different disease stages.

For early-stage CAVD characterized by ECM disorganization and progressive stiffening without massive calcification, matrix-stabilizing modalities are preferred. As a typical in vitro physical therapy modality, ultrasound has been well validated for its efficacy in accelerating fracture healing and skin regeneration^99^^,^^133^, ^134^, ^135^. Similarly, targeted mechanomedicine-based physical therapy can modulate the biological functions of VICs and VECs, suppress their abnormal phenotypic differentiation and pathological ECM stiffening, and thereby delay or even reverse the progressive pathological process of CAVD. Specifically, low-intensity pulsed ultrasound (LIPUS) exerts therapeutic effects mainly through cytoskeletal remodeling-mediated mechanotransduction, normalizing the aberrant phenotype of valvular interstitial cells without causing tissue damage.^136^^,^^137^ While ultrasound operates via an in vitro intervention mode, light-mediated therapy offers a highly promising in vivo physical treatment regimen for moderate-to-advanced CAVD. Existing studies have confirmed that light irradiation technology enables in situ localized crosslinking and stiffening of ECM proteins in ex vivo tissues. Notably, light-based modalities enable bidirectional mechanical modulation: targeted photo-crosslinking reinforces the disorganized fibrosa layer to halt progressive stiffening, while controlled photo-degradation selectively softens focal calcified plaques via localized ECM remodeling. Furthermore, relying on minimally invasive cardiovascular interventional techniques, this strategy can be extended to in vivo applications, achieving precise targeted crosslinking and softening of ECM within the diseased aortic valve region in living organisms. Integrated with endovascular light delivery catheters, the procedure can be performed under fluoroscopic guidance without open surgery, providing a minimally invasive middle-ground option for patients not yet indicated for valve replacement. This provides an innovative paradigm for the clinical management of CAVD and exhibits substantial translational application potential.^138^

**Third, mechanomedicine-guided intraoperative treatment decision-making.** The integration of computational fluid dynamics (CFD) and patient-specific anatomical modeling—cornerstone tools of mechanomedicine—may revolutionize personalized surgical planning for complex valvular heart diseases^139^, ^140^, ^141^. For valvuloplasty, it further guides surgical planning, including how to reshape the native valve, whether an annuloplasty ring (or prosthetic annulus) is required, and what ring size should be selected, so that the remodeled valve can better approximate the physiological mechanics of a normal human valve.^142^^,^^143^ This optimization reduces surgical duration and improves long-term patient outcomes, such as reduced reoperation rates.

Beyond surgical valvuloplasty, this biomechanical simulation framework also provides critical support for transcatheter aortic valve replacement (TAVR) planning. Preoperative CFD-finite element analysis (FEA) coupled simulation enables precise prosthetic valve sizing and prediction of optimal implantation depth, allowing clinicians to anticipate and mitigate high-risk complications such as paravalvular leakage, coronary obstruction and conduction block. Post-procedural hemodynamic simulation further predicts left ventricular unloading efficiency and long-term cardiac function recovery, offering objective indicators for prognostic evaluation. In addition, emerging intraoperative real-time elastography enables immediate assessment of leaflet stress distribution after repair or replacement, allowing on-table adjustment to achieve optimal biomechanical outcomes.^144^

**Fourth, mechanomedicine-optimized bioprosthetic valve development**. Integrating fluid mechanics and mechanobiology principles to refine bioprosthetic valve design—including tailoring annulus geometry to match the patient's individual aortic root dimensions for reducing paravalvular leakage and asymmetric flow, and optimizing leaflet design (e.g., trileaflet configuration, thickness gradient, and material stiffness) to mimic the compliance and shear stress distribution of native valves, thereby minimizing leaflet fatigue and calcification risk—and incorporating mechanomedicine-derived insights into CAVD pathogenesis (such as embedding targeted sustained-release drugs, e.g., anti-calcific or anti-inflammatory agents, within the valve structure), this integrated strategy holds the potential to prolong the service life of bioprosthetic valves. By addressing abnormal hemodynamic stress, valvular calcification, and pathological remodeling driven by mechanical dysregulation simultaneously, it aims to overcome the key limitations of current bioprosthetic valves.

At the functional modification level, two additional mechanomedicine-inspired strategies further enhance valve durability. First, mechano-mimetic surface modification with engineered micro/nanotopography and tuned substrate stiffness promotes rapid endothelialization after implantation, forming a native-like endothelial barrier that reduces thrombogenicity and inhibits subendothelial calcification.^120^ Second, computational fatigue life simulation based on long-term cyclic loading enables preclinical prediction of valve mechanical durability, shortening the development cycle of new prosthetic products and reducing the risk of late structural valve deterioration.^145^

**Fifth, mechanomedicine-driven engineering of tissue-engineered heart valves (TEHVs)**. Mechanical valves offer long durability but require lifelong anticoagulation, whereas bioprosthetic valves obviate the need for lifelong anticoagulation yet are prone to structural valve deterioration (SVD). Additionally, for pediatric and young patients requiring valve replacement, prosthetic valves fail to grow synchronously with the patient, necessitating reoperation. The Ross procedure is highly invasive; moreover, using the pulmonary valve as an aortic valve substitute leads to substantial hemodynamic alterations, making reoperation inevitable.^146^ Although allograft valves are available, their supply is extremely limited.^147^^,^^148^ To address these limitations, personalized TEHV fabrication can be performed based on the patient's native aortic sinus anatomy, including patient-specific scaffold design derived from 3D imaging of the aortic sinus to ensure anatomical compatibility and optimal hemodynamic performance, seeding autologous cells preconditioned with mechanical stimulation (e.g., pulsatile flow, cyclic stretching, shear stress) to induce their differentiation toward valvular cell phenotypes, culturing the construct in bioreactors that simulate the mechanical environment of native aortic valves to promote the synthesis and ordered arrangement of extracellular matrix (e.g., collagen fiber alignment along mechanical stress lines), and utilizing biodegradable scaffold materials (e.g., polycaprolactone/gelatin composites) with degradation rates matching tissue regeneration to enable TEHV remodeling driven by in vivo hemodynamic forces.^149^^,^^150^

Beyond in vitro prefabricated TEHVs, in situ induced valve regeneration represents another important direction driven by mechanomedicine: by implanting biodegradable scaffolds with optimized hemodynamic design into the aortic valve position, the body's own cells are recruited and guided by local mechanical signals to assemble into functional valve tissue, avoiding the complexity and immune risks of in vitro cell culture.^151^^,^^152^ For pediatric patients, growth-adaptable TEHV design with anisotropic mechanical properties allows the valve to gradually remodel and grow synchronously with the child's cardiovascular system under physiological hemodynamic forces, fundamentally solving the problem of repeated reoperations caused by size mismatch.^153^ This strategy holds the potential to reduce immune rejection post-implantation, extend valve lifespan, and potentially facilitate in vivo valve remodeling and growth. Eventually, the scaffold undergoes complete biodegradation, achieving full in situ valve regeneration.

**Sixth, mechanomedicine-enabled early diagnosis of CAVD**. The early pathological hallmark of CAVD is valvular thickening and stiffening, which may not be accompanied by impaired aortic valve opening and closure. Given that ultrasound-based elastography (a mechanomedicine-derived technique) assesses organ stiffness for liver cirrhosis diagnosis, and MRI elastography analyzes brain tissue stiffness for Alzheimer's disease diagnosis, developing analogous valvular elastography techniques would facilitate early CAVD diagnosis by detecting pre-symptomatic stiffness changes.^154^^,^^155^

Further extending from structural stiffness detection to multi-dimensional mechanical phenotyping, integrated multimodal mechanical imaging—combining valvular elastography, 4D flow MRI-derived wall shear stress mapping, and finite element-based stress distribution analysis—enables comprehensive quantification of the valvular biomechanical state at the subclinical stage. On this basis, mechanics-driven risk stratification models can be constructed to identify high-risk progressors among patients with early aortic sclerosis, guiding precise timing of intervention and follow-up strategies, and shifting the clinical management window from late-stage valve replacement to early disease modification.

Collectively, these advancements—spanning drug development, physical therapy, surgical decision-making, prosthetic design, tissue engineering, and early diagnosis—highlight the transformative potential of mechanomedicine in addressing unmet clinical needs in CAVD management, while underscoring the need for further research to resolve technical and translational challenges. Moving forward, the integration of artificial intelligence and high-throughput biomechanical testing will further accelerate the translation of mechanomedicine innovations from bench to bedside.

## 3 Future research directions

Over the next five years, to drive the in-depth advancement and clinical translation of mechanobiological research on CAVD, multidisciplinary strengths shall be integrated for collaborative breakthroughs, with focused efforts on addressing four core scientific challenges and technical bottlenecks that currently constrain the development of this field.

**First, mechanomedicine-based standardized in vivo biomechanical parameter reference database**. As stated previously, hemodynamic indices and valvular stiffness parameters reported in existing studies show marked heterogeneity, with discrepancies across different reports reaching an order of magnitude. Furthermore, well-validated consensus reference ranges for physiological and pathological conditions have not yet been established. Subsequent work shall integrate in vivo human imaging data, ex vivo experimental measurements of tissue biomechanics, and high-precision computational simulation data to build an open and shared standardized reference database. The database shall cover age-stratified reference values for healthy populations, biomechanical signatures across all stages of CAVD, as well as unified measurement methodologies and result reporting specifications. This standardized framework will enable cross-study comparability of research findings, significantly improve experimental reproducibility, and establish an objective quantitative benchmark for both basic mechanistic exploration and clinical disease assessment.

**Second, mechanomedicine-enabled high-fidelity in vitro model systems with mechanical decoupling**. Existing bioreactor platforms can hardly achieve independent and precise regulation of pressure, shear stress, and cyclic stretch while faithfully recapitulating the characteristics of native pulsatile blood flow. Meanwhile, research conclusions on matrix stiffness are significantly affected by the two-dimensional/three-dimensional culture modality of VICs, leading to insufficient consistency of findings. Next-generation bioreactor systems shall realize independent, tunable and precise control of various mechanical stimuli under physiological pulsatile flow conditions, paired with a standardized three-dimensional co-culture system of VICs and VECs to restore the layered microstructure and cellular microenvironment of native valve leaflets. Such advanced model systems will help researchers clarify the independent role of each mechanical stimulus in the onset and progression of CAVD, and enable high-precision quantitative analysis of the dose–response relationship between mechanical input and cellular phenotypic transition.

**Third, mechanomedicine-derived therapeutic strategies for in vivo validation and translation.** Although mechanistic studies have identified a series of promising mechanotherapeutic targets, supporting evidence is mostly limited to in vitro cellular systems and small animal models, lacking high-level in vivo evidence. Over the next five years, research priorities will focus on three aspects: first, to establish large-animal CAVD models with short modeling cycles and stable phenotypes, providing a reliable platform for in vivo efficacy validation of targeted therapies; second, to screen and validate specific biomarkers for aortic valve disease based on large cohorts of clinical specimens, laying a molecular foundation for multiplex precise targeted drug delivery; third, to develop clinically feasible local delivery technologies for mechanically targeted drugs, such as transcatheter targeted drug delivery systems for aortic valve leaflets, to provide a technical pathway for precise valvular drug administration.

**Fourth, mechanomedicine-empowered noninvasive valvular biomechanics assessment techniques.** The early pathological hallmark of CAVD is elevated valvular stiffness at the subclinical stage, which occurs prior to detectable hemodynamic stenosis. However, conventional clinical imaging modalities can only evaluate valve morphology and transvalvular flow velocity. Core biomechanical parameters such as regional valvular stiffness and wall shear stress distribution can only be estimated indirectly via computational simulation, and real-time, in vivo direct quantitative detection remains unavailable. Over the next five years, integrated innovation and clinical validation of multimodal imaging techniques including ultrasound elastography, magnetic resonance elastography and four-dimensional flow magnetic resonance imaging (4D flow MRI) shall be promoted to achieve noninvasive, accurate and reproducible quantification of key valvular biomechanical parameters. Integrating these biomechanical biomarkers into existing clinical risk stratification models will facilitate early disease identification, precise prognostic staging, and real-time dynamic monitoring of treatment response. The achievement of the above objectives depends on sustained and structured interdisciplinary collaboration among cardiac surgeons, molecular biologists, biomechanical engineers and imaging scientists. Breaking through these technical and translational barriers will fully unlock the application potential of mechanobiomedicine, and transform the CAVD diagnosis and treatment paradigm from end-stage surgical replacement to early mechanism-driven precise intervention.

## 4 Conclusion

Mechanobiology plays an indispensable role in the onset and progression of CAVD, with hemodynamic forces and ECM stiffness serving as core modulators of valvular cell phenotypes and pathological extracellular matrix remodeling. A key conceptual contribution of this review is the systematic delineation of a dichotomous pathogenetic role for mechanobiological factors across disease etiologies: in congenital conditions such as BAV malformation and Marfan syndrome, abnormal hemodynamics or matrix stiffness act as initiating drivers that directly trigger disease onset; whereas in most acquired CAVD cases secondary to hyperlipidemia, inflammation, or metabolic disorders, mechanobiological alterations function as secondary amplifiers that perpetuate disease progression via a self-reinforcing positive feedback loop. This framework resolves longstanding ambiguity regarding the causal role of mechanics in CAVD and provides a theoretical basis for etiology-stratified mechanotherapeutic strategies.

Building on these mechanobiological insights, the emerging paradigm of mechanomedicine holds immense promise for revolutionizing CAVD management, offering transformative solutions spanning early noninvasive diagnosis, novel mechanotransduction-targeted drug development, computational fluid dynamics-guided personalized surgical planning, and mechanically optimized advanced valve engineering. However, translating these prospects into clinical practice requires overcoming substantial challenges, including the lack of standardized biomechanical parameter reference values, limited access to high-quality clinical specimens, technical barriers in decoupling complex interdependent mechanical cues, and the urgent need to strengthen structured interdisciplinary collaboration among cardiac clinicians, molecular biologists, and biomechanical engineers.

## CRediT authorship contribution statement

**Yefan Jiang:** Conceptualization. **Yuqiu He:** Methodology. **Qiong Jia:** Writing – review & editing, Writing – original draft. **Si Chen:** Supervision, Resources. **Youhua Tan:** Supervision, Resources, Project administration. **Yongfeng Shao:** Visualization, Validation, Supervision.

## Ethics approval and consent to participate

This article is a review and ethics approval is not necessary.

## Availability of data and material

The data are available from the corresponding author on reasonable request.

## Declaration of competing interest

The authors declare that they have no known competing financial interests that could have appeared to influence the work reported in this paper.
