# Supplementary material for: From biomechanical mechanisms to clinical reasoning: Deciphering mechanobiological drivers of aortic valve calcification for precision therapy
Source: Mechanobiol Med. 2026 Aug 22;4(3):100208. doi: 10.1016/j.mbm.2026.100208 (PMC13545724; doi:10.1016/j.mbm.2026.100208)
Supplement: Supplementary file 1 [file mmc1.docx]

|  | **Names of first author** | **Measurement methods** | | | | | **Normal aortic valve values (mmHg)** | **CAVD aortic valve values (mmHg)** | | | |  |
| --- | --- | --- | --- | --- | --- | --- | --- | --- | --- | --- | --- | --- |
| Pressure | Damien Garcia [1] | In vivo | | | | | - | intra-aortic pressure: systolic phase: 98±12; diastolic phase: 60±10  Left ventricular pressure: end-systole phase: 83±12; end-diastolic phase: 13±7; peak systolic phase: 163±24 | | | |  |
|  | Hoda Maleki [2] | In vitro simulation using a bioreactor (HR:70 bpm; CO: 5L/min,BP: 120/80 mmHg | | | | | Peak left ventricle pressure: 122.8 | As the degree of aortic valve stenosis increases, the left ventricular pressure also increases, reaching up to 150-180 | | | |  |
|  | Neelakantan Saikrishnan [3] | In vitro simulation using a bioreactor^＊^ (HR:70 bpm; CO: 5L/min,BP: 100 mmHg | | | | | 128.88 | 160 | | | |  |
|  | **Names of first author** | **Measurement methods** | | | | | **Normal aortic valve values (dyn/cm^2^)** | **CAVD aortic valve values (dyn/cm^2^)** | | | |  |
| Shear stress | Dana M.Stevenson [4] | In vitro simulation using a bioreactor | | | | | 20-27 (orifice area of 5 cm²)(Ventricular side) | 60-65 (orifice area of 3.5 cm²)  75-80 (orifice area of 1.6 cm²)  330-335 (orifice area of 1.6 cm²) | | | |  |
|  | Philippe Sucosky[5] | Not Mentioned | | | | | Ventricular side: 0-80；  aortic side:-8-10 | - | | | |  |
|  | Choon Hwai Yap [6, 7] | In vitro simulation using a bioreactor (BP: 120/80 mmHg; Constant HR: 70 bpm, variable CO: 1.89, 3.01, 4.34, 4.76L/min; or Constant CO: 2.75, 3.85, 4.95L/min) | | | | | -5-20(Ventricular side) | - | | | |  |
|  | Choon Hwai Yap [8] | Using the ascending aortic volumetric flow rates acquired through PC-MRI based on the theoretical model (In vitro simulation of normal aortic valves using a bioreactor with HR: 70bpm; CO: 4.5-5L/min and BP:120/80mmHg) | | | | | -41-50—64-71(Ventricular side) |  | | | |  |
|  |  |  |  |  |  |  | -35-38—77-92(Ventricular side) |  | | | |  |
|  | Santanu Chandra [9] | FEA | | | | |  | non-fused valves | | fused valves | |  |
|  |  |  |  |  |  |  |  | Moderate calcification | Severe calcification | Moderate calcification | Severe calcification |  |
|  |  | Systolic phase | | Aortic side | | Tip | -5.4-3.06 | -12.32-5.04 | -11.06-0.85 | -5.04-4.75 | -5.74-4.75 | |
|  |  |  |  |  |  | Middle | -1.67-0.87 | -3.27-2 | -1.03-0.61 | -1.87-0.61 | -1.87-0.61 | |
|  |  |  |  |  |  | Base | -1.39-0 | -2.2-0 | -1.39-0 | -5.04-1.18 | -3.29-1.08 | |
|  |  |  |  | Ventricular side | | Tip | 11.58-31.23 | 23.6-65.26 | 0-18.25 | 26.32-90.53 | 26.32-55.79 | |
|  |  |  |  |  |  | Middle | 5.61-40 | 5.61-103.51 | 5.61-36.84 | -22.11-54.04 | -22.11-40 | |
|  |  |  |  |  |  | Base | 13.68-43.86 | 0-88.07 | 0-22.46 | 0-15.09 | 0-5.26 | |
|  | Choon Hwai Yap [3, 10] | In vitro simulation using a bioreactor^＊^ (HR:70 bpm; CO: 5L/min,BP: 100 mmHg | | | | | Tricuspid aortic valve | Bicuspid aortic valve  (fused valve) | | Bicuspid aortic valve  (non-fused valve) | |  |
|  |  | Aortic side | | | | Base | - | 13 | | - | | |
|  |  | Ventricular side | | | |  | 20 | 19.9 | | 36 | | |
|  | **Names of first author** | **Measurement methods** | | | | | **Normal aortic valve values (%)** | **CAVD aortic valve values (%)** | | | |  |
| Stretching force | Choon Hwai Yap/Balachandran K [11, 12] | In vitro simulation using a bioreactor with dual-camera photogrammetry (BP:80/120 mmHg, 120/160 mmHg and 150/190 mmHg) | | | | | 9-11% (circumferential);  13-25% (radial) | Under high blood pressure: 15% (circumferential), 13-31% (radial) | | | |  |
|  | Ankush Aggarwal [13] | Human data: Preoperative echocardiography, postoperative imaging of the aortic valves in a flattened state | | | | | 20% increased (open state ex vivo VS in vivo )25% increased (closed state VS open state) |  | | | |  |
|  | **Names of first author** | **Measurement methods** | | | | | **Normal aortic valve values (kPa)** | **CAVD aortic valve values (kPa)** | | | |  |
| Combined | Guangzhou Li [14] | FEA | | | | | Tricuspid aortic valve | Bicuspid aortic valve  (fused valves) | | Bicuspid aortic valve  (non-fused valves) | |  |
|  |  | Ventricular side | 25mmHg^＊＊^ | | Systolic phase | Tip | 116 | 100 | | 186 | | |
|  |  |  |  |  |  | Base | 124 | 160 | | 148 | | |
|  |  |  |  |  | Diastolic phase | Tip | 60 | 102 | | 52 | | |
|  |  |  |  |  |  | Base | 45 | 54 | | 50 | | |
|  |  |  | 50mmHg^＊＊^ | | Systolic phase | Tip | 251 | 164 | | 226 | | |
|  |  |  |  |  |  | Base | 174 | 270 | | 301 | | |
|  |  |  |  |  | Diastolic phase | Tip | 115 | 195 | | 73 | | |
|  |  |  |  |  |  | Base | 105 | 100 | | 98 | | |

Supplementary Table 1: **In CAVD, changes in shear stress, pressure, and stretching forces experienced by the aortic valves**

Blood pressure: BP; HR: heart ratio; CO: cardiac output; Finite element analysis: FEA

^＊^left and right coronary arteries stitched together to create a bicuspid stenosis model

^＊＊^across valve pressure difference

|  | **Names of first author** | **Range (mmHg)** | **Pulsatile(Yes/No)** | **Bioreactor Type (Self-developed/Commercial)** | **Research subjects** | **Conclusions** |
| --- | --- | --- | --- | --- | --- | --- |
| Pressure | Michael S. Sacks [15] | 0, 1, 2, 4, 20, 60, 90 | No | Self-developed | Porcine aortic valves | Pressure can regulate the alignment of fibers in the ECM |
|  | Yun Xing [16] | 100, 140, 170 | No | Self-developed | Porcine aortic valves | Pressure can regulate ECM composition |
|  | Yun Xing [17] | 100, 140, 170 | 0.5, 1.167, and 2 Hz | Self-developed | Porcine aortic valves | Pressure can regulate ECM composition, VICs’ proliferation；  Aortic valves’ response to pressure depends on its magnitude and frequency |
|  | Suchtra Konduri [18] | 120/80 | Around 1.25Hz | Self-developed | Porcine aortic valves | Physiological pressure maintains vavular ECM composition, cellular phenotype, and shape |
|  | Hsiao-Ying Shadow Huang [19] | 0, 1, 2, 4, 20, 60, 90 | No | Self-developed | Porcine aortic valves | Pressure can regulate the alignment of fibers in the ECM and VICs’ nuclear shape |
|  | Erinn M. Joyce [20] | 0, 1, 2, 4, 10, 20, 60, 90 | No | Self-developed | Porcine aortic valves | Pressure can regulate the alignment of fibers in the ECM |
|  | Choon Hwai Yap [11] | 120/80 (physiological), 160/120 (hypertensive) and 190/150 (severe hypertensive) | 1.17Hz | Self-developed | Porcine aortic valves | Pressure can regulate the stretching of the valves |
|  | James N. Warnock [21] | 80, 120 | 1 Hz | Self-developed | Porcine VICs | Pressure can regulate inflammatory response |
|  | Valtresa Myles [22] | 80 (physiological)，120 (hypertensive) | No | Self-developed | Porcine aortic valves | Pressure can regulate ECM composition, valvular elasticity, and cellular stiffness |
|  | **Names of first author** | **Range (dynes/cm^2^)** | **Pulsatile(Yes/No)** | **Bioreactor Type (Self-developed/Commercial)** | **Research subjects** | **Conclusions** |
| Shear stress | Jonathan T. Butcher [23] | 20 | No | Self-developed | Porcine VECs | Shear stress can regulate VECs' shape |
|  | Jonathan T. Butcher [24] | 20 (pathological) | No | Self-developed | Porcine VECs | Shear stress can regulate VECs’ activation and inflammatory response |
|  | Jonathan T. Butcher [25] | 20 | No | Self-developed | Porcine VICs and VECs | Shear stress can regulate VICs’ phenotype, proliferation and apoptosis and protein secretion (regulating ECM composition); VECs can inhibit shear stress-induced phenotypic differentiation of VICs |
|  | Philippe Sucosky [5] | ventricular side: 0-80 | Around 1.25Hz | Self-developed | Porcine aortic valves | Shear stress can regulate inflammatory response |
|  |  | aortic side: −8-10 | Around 1.25Hz |  |  |  |
|  | Casey J. Holliday [26] | ventricular side: 20 |  | Self-developed | Human VECs | Shear stress can regulate VECs’ shape and activation |
|  |  | aortic side: -5-5 | 1Hz |  |  |  |
|  | Ling Sun [27] | Ventricular side: 0-39, 0-11, 0-78; Aortic side: -1.1-0.4, -4.5-1.1, -0.4-1.8 ^＊^ | 1.167Hz | Self-developed | Porcine aortic valves | Shear stress can regulate VECs’ activation and ECM composition |
|  | Ling Sun [28] | Normal parameters: 0-83 (ventricular side)，-10-10 (aortic side)；  Abnormal parameters were half or twice normal parameters | 1.167Hz | Self-developed | Porcine aortic valves | Shear stress can regulate VECs’ activation and ECM composition |
|  | Gretchen J. Mahler [29] | Laminar: 2, 10, 20；Oscillatory  : -2-2, -10-10, -20-20 | Laminar: No；Oscillatory: 1Hz | Self-developed | Porcine VECs | Shear stress can regulate VECs’ EMT process and inflammatory response |
|  | Jack M. Heath/Joan Fernandez Esmerats [30, 31] | Laminar: 20; Oscillatory: -5-5 | 1Hz | Self-developed | Human and Porcine VECS | Shear stress can regulate ECM composition |
|  | Guoheng Zhong [32] | Laminar: 12，Oscillatory: 0.5±4 | Not mentioned | Ibidi | Human VICs | Shear stress can regulate VICs’ phenotype |
|  | **Names of first author** | **Range(%)** | **Pulsatile(Yes/No)** | **Bioreactor Type (Self-developed/Commercial)** | **Research subjects** | **Conclusions** |
| Stretching force | Kartik Balachandran [33] | 15 | 1.167Hz | Self-developed | Porcine aortic valves | Stretching can regulate ECM composition and VICs’ phenotype |
|  | W. David Merryman [34] | 15 | 1Hz | Self-developed | Porcine aortic valves | Stretching can regulate ECM composition and VICs’ phenotype which is synergistic with TGF-β1 |
|  | Kartik Balachandran [35] | 10 (physiological), 15 , and 20 | 1.167Hz | Self-developed | Porcine aortic valves | Stretching can regulate ECM composition and VICs’ proliferation and apoptosis, |
|  | Kathryn E. Smith [36] | 0, 5, 10, 15 (physiological) and 20 | 1Hz | Flexercell 4000T | Porcine VICs | Stretching can regulate inflammatory response |
|  | Kartik Balachandran [37] | 10 (physiological)，15^＊＊^ | 1.167Hz | Self-developed | Porcine aortic valves | Stretching in conjunction with calcium and phosphate deposition model/high phosphate model/TGF-β1 can regulate CAVD calcification; Stretching can regulate VICs’ apoptosis |
|  | Patrick Thayer [38] | 10 (physiological)，15^＊＊＊^ | 1.167Hz | Self-developed | Porcine aortic valves | Stretching can regulate VICs’ phenotype; The pressure affects how stretching regulates VICs’ phenotype |
|  | Kartik Balachandran [39] | 10 | 1.167Hz | Self-developed | Porcine aortic valves | Stretching can regulate ECM composition and VICs’ proliferation |
|  | Kartik Balachandran [40] | 10 (physiological)，15 | Around 1.11Hz | Self-developed | Porcine aortic valves | Stretching can regulate ECM composition and VICs’ proliferation |
|  | Joshua D Hutcheson [41] | 10, 20 | 1Hz | Flexcell 4000 | Porcine VICs | Stretching can regulate VICs’ apoptosis |
|  | Russell A. Gould [42] | 20 | 1Hz | Self-developed | Porcine VICs | Stretching can regulate VICs’ phenotype and cytoskeletal alignment and the regulation by stretching is directional |
|  | Charles I. Fisher [43] | 5, 10, 15 and 20 | 0.75Hz | Flexcell-4000 | Porcine VICs | Stretching in conjunction with TGF-β1 can regulate VICs’ phenotype and promotes the formation of calcium nodules |
|  | Christopher Moraes [44] | 3, 12 | 1Hz | Self-developed | Porcine VICs | Stretching can regulate VICs’ phenotype |
|  | Zannatul Ferdous [45] | 5 , 10 (physiological), and 15 | 1Hz | Self-developed | Human VICs | Stretching can regulate ECM composition and VICs’ proliferation |
|  | Joseph Chen [46] | 10 | 1Hz | Flexcell-4000 | Mice VICs | Stretching can regulate VICs’ proliferation |
|  | Ngoc Thien Lam [47] | 10，20 | 1Hz | Self-developed | Porcine aortic valves | Stretching can regulate VICs’ shape |
|  | Scott A. Metzler [48] | 15 | 1Hz | Not mentioned | Porcine VECs | Stretching can regulate ECM composition and the live cell ratio of VECs |
|  | Md Tausif Salim [49] | 10 (physiological), 15 | Not mentioned | Self-developed | Porcine aortic valves | Calcium phosphate deposition model affects how stretch regulates VICs’ phenotype |
|  | Md Tausif Salim [50] | 15 | 1Hz | Self-developed | Porcine aortic valves | Stretching promotes the formation of calcium nodules |
|  | Toni M. West [51] | 10 | 1Hz | Self-developed | Porcine VICs | Stretching can regulate the area of VICs and nuclei, as well as the polymerization of α-sma and vimentin |

Supplementary Table 2 | **Summary of the hemodynamic parameters used in published studies**

^＊^Ventricular side: 0-39 (base of tricuspid aortic valves), 0-11 (base of fused valves in bicuspid aortic valves), 0-78 (base of non-fused valves in bicuspid aortic valves); Aortic side: -1.1-0.4 (base of tricuspid aortic valves), -4.5-1.1(base of fused valves in bicuspid aortic valves), -0.4-1.8(base of non-fused valves in bicuspid aortic valves)

^＊＊^combined with calcium and phosphate deposition model and TGF-β1

^＊＊＊^(combined with pressure：120/80mmHg (physiological)，140/100mmHg (hypertensive))

| **Names of first author** | **Measurement methods** | **Subject** | **Normal aortic valve values (ka)** | **CAVD aortic valve values (ka)** | | |
| --- | --- | --- | --- | --- | --- | --- |
| Ruogang Zhao [52] | Micropipette aspiration | Porcine | Aortic side: 1.69 (0.59-4.54)；Ventricular side: 1.14 (0.12-3.04) | - | | |
| M.K. Sewell-Loftin [53] | Atomic force microscopy | Mouse | 2-month-old wild mice: 470; 17-month-old wild mice: 580 | 20-month-old Apoe^-/-^ mice: 760; 20-month-old Notch1^+/-^: 1350 | | |
|  | Atomic force microscopy | Porcine | Ventricularis: 1130; Spongiosa: 650；Fibrosa: 1930 |  | | |
| Huan Wang [54] | Synthesizing previous studies | Human | 0.8-8 | 17-37 | | |
| Hoda Maleki [55] | Bench-top microindentation system | Porcine^＊^ | Aortic side: 5.00±1.78；Ventricular side: 2.61±0.63 |  | Lesion on the aortic side of calcified valves | Non-lesion on the aortic side of calcified valves |
|  |  |  |  | Non-coronary cusp | 1.76 | 4.92 |
|  |  |  |  | Left coronary cusp | 2.12 | 4.64 |
|  |  |  |  | Right coronary cusp | 1.76 | 4.06 |
|  |  |  |  | Mean | 1.87±0.46 | 4.56±0.95 |

Supplementary Table 3 | **Summary of the reported stiffness of normal and CAVD aortic valves**

^＊^normal and early stages of aortic valve disease

| **Names of first author** | **Range**  **(ka)** | **Composition** | **Peptides adhered** | **Culture Model**  **（2D/3D）** | **Cell/Tissue Type** | **Conclusions** |
| --- | --- | --- | --- | --- | --- | --- |
| Cindy Ying Yin Yip [56] | 2, 6 | Collagen | Collagen | 2D | Porcine VICs | Regulation of VIC phenotype by ECM stiffness is related to the calcium and phosphate deposition; ECM stiffness can regulate VICs’ proliferation and apoptosis |
| April M. Kloxin [57] | 7, 15, 32 | PEG | Fibronectin | 2D | Porcine VICs | ECM stiffness can regulate VICs’ phenotype |
| Jan-Hung Chen [58] | 3, 11, 22, 50, 144 | Acrylamide and bis- acrylamide | Collagen | 2D | Porcine VICs | ECM stiffness can regulate VICs’ phenotype |
| Huan Wang [54] | 7.3×10^6^ | PEG | RGD | 2D | Porcine VICs | ECM stiffness can regulate VICs’ phenotype |
| Chelsea M. Kirschner [59] | 3, 15 | PEG | RGD | 2D | Porcine VICs | ECM stiffness can regulate VICs’ phenotype; Regulation of VICs’ phenotype by ECM stiffness is related to the surface smoothness |
| Sarah T. Gould [60] | 3, 27 | PEG | RGD | 2D | Porcine VICs and VECs | ECM stiffness can regulate VICs’ phenotype; VECs can inhibit ECM stiffness-induced phenotypic differentiation in VICs |
| Liezl Rae Balaoing [61] | Not metioned^＊^ | PEG | RKR and RGD | 2D | Porcine VECs | ECM stiffness and composition can both regulate VECs’ activation |
| Hao Ma [62] | 2, 4, 6, 10 | PEG | RGD | 2D | Porcine VICs | ECM stiffness can regulate VICs’ phenotype and shape; Regulation of VICs’ phenotype by ECM stiffness is related to the uniformity of ECM stiffness |
| Sudip Dahal [63] | 3.05, 8.42, 37.61 | Collagen I | CS, DS and HA | 3D | Porcine VECs | ECM stiffness can regulate VECs’ EMT process and this process is affected by ECM composition |
| Aileen Zhong [64] | 5, 50 | Sylgard 527 silicone | Collagen I | 2D | Porcine VECs | ECM stiffness can regulate VECs’ EMT process |
| Alex Khang [65] | 2.5, 5, 10 | PEG | RGD | 3D | Porcine VICs | ECM stiffness can regulate VICs’ phenotype |
| Cierra J. Walker [66] | 1.7, 4.5 | PEG | RGD | 2D | Porcine VICs | ECM stiffness can regulate VICs’ phenotype and shape |
| Sudip Dahal [67] | 3.05, 8.42, 37.61 | Collagen I | CS, DS and HA | 3D | Porcine VICs and VECs | ECM stiffness can regulate VICs’ phenotype which is inhibited by VECs; ECM stiffness can regulate formation of calcium nodules in VICs which is inhibited by VECs; ECM stiffness can regulate VICs’ proliferation; ECM composition can affect the regulation of ECM stiffness on VICs’ phenotype, formation of calcium nodules and proliferation |
| Dilara Batan [68] | 1.5, 15.2 | PEG | RGD | 2D | Porcine VICs | ECM stiffness can regulate VICs’ phenotype and proliferation |
| Cierra J. Walker [69] | 5, 15 | PEG | RGD | 2D | Porcine VICs | ECM stiffness can regulate VICs’ phenotype and shape |

Supplementary Table 4 | **Summary of** **the stiffness values used in published studies**

**References:**

[1] D. Garcia, P.J. Barenbrug, P. Pibarot, A.L. Dekker, F.H. van der Veen, J.G. Maessen, J.G. Dumesnil, L.G. Durand, A ventricular-vascular coupling model in presence of aortic stenosis, Am J Physiol Heart Circ Physiol 288(4) (2005) H1874-84.

[2] H. Maleki, S. Shahriari, L.G. Durand, M.R. Labrosse, L. Kadem, A metric for the stiffness of calcified aortic valves using a combined computational and experimental approach, Med Biol Eng Comput 52(1) (2014) 1-8.

[3] N. Saikrishnan, C.H. Yap, N.C. Milligan, N.V. Vasilyev, A.P. Yoganathan, In vitro characterization of bicuspid aortic valve hemodynamics using particle image velocimetry, Ann Biomed Eng 40(8) (2012) 1760-75.

[4] D.M. Stevenson, A.P. Yoganathan, F.P. Williams, Numerical simulation of steady turbulent flow through trileaflet aortic heart valves--II. Results on five models, J Biomech 18(12) (1985) 909-26.

[5] P. Sucosky, K. Balachandran, A. Elhammali, H. Jo, A.P. Yoganathan, Altered shear stress stimulates upregulation of endothelial VCAM-1 and ICAM-1 in a BMP-4- and TGF-beta1-dependent pathway, Arterioscler Thromb Vasc Biol 29(2) (2009) 254-60.

[6] C.H. Yap, N. Saikrishnan, G. Tamilselvan, A.P. Yoganathan, Experimental measurement of dynamic fluid shear stress on the aortic surface of the aortic valve leaflet, Biomech Model Mechanobiol 11(1-2) (2012) 171-82.

[7] C.H. Yap, N. Saikrishnan, G. Tamilselvan, A.P. Yoganathan, Experimental technique of measuring dynamic fluid shear stress on the aortic surface of the aortic valve leaflet, J Biomech Eng 133(6) (2011) 061007.

[8] C.H. Yap, N. Saikrishnan, A.P. Yoganathan, Experimental measurement of dynamic fluid shear stress on the ventricular surface of the aortic valve leaflet, Biomech Model Mechanobiol 11(1-2) (2012) 231-44.

[9] S. Chandra, N.M. Rajamannan, P. Sucosky, Computational assessment of bicuspid aortic valve wall-shear stress: implications for calcific aortic valve disease, Biomech Model Mechanobiol 11(7) (2012) 1085-96.

[10] C.H. Yap, N. Saikrishnan, G. Tamilselvan, N. Vasilyev, A.P. Yoganathan, The congenital bicuspid aortic valve can experience high-frequency unsteady shear stresses on its leaflet surface, Am J Physiol Heart Circ Physiol 303(6) (2012) H721-31.

[11] C.H. Yap, H.S. Kim, K. Balachandran, M. Weiler, R. Haj-Ali, A.P. Yoganathan, Dynamic deformation characteristics of porcine aortic valve leaflet under normal and hypertensive conditions, Am J Physiol Heart Circ Physiol 298(2) (2010) H395-405.

[12] K. Balachandran, P. Sucosky, A.P. Yoganathan, Hemodynamics and mechanobiology of aortic valve inflammation and calcification, Int J Inflam 2011 (2011) 263870.

[13] A. Aggarwal, A.M. Pouch, E. Lai, J. Lesicko, P.A. Yushkevich, J.H. Gorman Iii, R.C. Gorman, M.S. Sacks, In-vivo heterogeneous functional and residual strains in human aortic valve leaflets, J Biomech 49(12) (2016) 2481-90.

[14] G. Li, N. Shen, H. Deng, Y. Wang, G. Kong, J. Shi, N. Dong, C. Deng, Abnormal mechanical stress on bicuspid aortic valve induces valvular calcification and inhibits Notch1/NICD/Runx2 signal, PeerJ 11 (2023) e14950.

[15] M.S. Sacks, D.B. Smith, E.D. Hiester, The aortic valve microstructure: effects of transvalvular pressure, J Biomed Mater Res 41(1) (1998) 131-41.

[16] Y. Xing, Z. He, J.N. Warnock, S.L. Hilbert, A.P. Yoganathan, Effects of constant static pressure on the biological properties of porcine aortic valve leaflets, Ann Biomed Eng 32(4) (2004) 555-62.

[17] Y. Xing, J.N. Warnock, Z. He, S.L. Hilbert, A.P. Yoganathan, Cyclic pressure affects the biological properties of porcine aortic valve leaflets in a magnitude and frequency dependent manner, Ann Biomed Eng 32(11) (2004) 1461-70.

[18] S. Konduri, Y. Xing, J.N. Warnock, Z. He, A.P. Yoganathan, Normal physiological conditions maintain the biological characteristics of porcine aortic heart valves: an ex vivo organ culture study, Ann Biomed Eng 33(9) (2005) 1158-66.

[19] H.Y. Huang, J. Liao, M.S. Sacks, In-situ deformation of the aortic valve interstitial cell nucleus under diastolic loading, J Biomech Eng 129(6) (2007) 880-89.

[20] E.M. Joyce, J. Liao, F.J. Schoen, J.E. Mayer, Jr., M.S. Sacks, Functional collagen fiber architecture of the pulmonary heart valve cusp, Ann Thorac Surg 87(4) (2009) 1240-9.

[21] J.N. Warnock, B. Nanduri, C.A. Pregonero Gamez, J. Tang, D. Koback, W.M. Muir, S.C. Burgess, Gene Profiling of Aortic Valve Interstitial Cells under Elevated Pressure Conditions: Modulation of Inflammatory Gene Networks, Int J Inflam 2011 (2011) 176412.

[22] V. Myles, J. Liao, J.N. Warnock, Cyclic pressure and angiotensin II influence the biomechanical properties of aortic valves, J Biomech Eng 136(1) (2014) 011011.

[23] J.T. Butcher, A.M. Penrod, A.J. Garcia, R.M. Nerem, Unique morphology and focal adhesion development of valvular endothelial cells in static and fluid flow environments, Arterioscler Thromb Vasc Biol 24(8) (2004) 1429-34.

[24] J.T. Butcher, S. Tressel, T. Johnson, D. Turner, G. Sorescu, H. Jo, R.M. Nerem, Transcriptional profiles of valvular and vascular endothelial cells reveal phenotypic differences: influence of shear stress, Arterioscler Thromb Vasc Biol 26(1) (2006) 69-77.

[25] J.T. Butcher, R.M. Nerem, Valvular endothelial cells regulate the phenotype of interstitial cells in co-culture: effects of steady shear stress, Tissue Eng 12(4) (2006) 905-15.

[26] C.J. Holliday, R.F. Ankeny, H. Jo, R.M. Nerem, Discovery of shear- and side-specific mRNAs and miRNAs in human aortic valvular endothelial cells, Am J Physiol Heart Circ Physiol 301(3) (2011) H856-67.

[27] L. Sun, S. Chandra, P. Sucosky, Ex vivo evidence for the contribution of hemodynamic shear stress abnormalities to the early pathogenesis of calcific bicuspid aortic valve disease, PLoS One 7(10) (2012) e48843.

[28] L. Sun, N.M. Rajamannan, P. Sucosky, Defining the role of fluid shear stress in the expression of early signaling markers for calcific aortic valve disease, PLoS One 8(12) (2013) e84433.

[29] G.J. Mahler, C.M. Frendl, Q. Cao, J.T. Butcher, Effects of shear stress pattern and magnitude on mesenchymal transformation and invasion of aortic valve endothelial cells, Biotechnol Bioeng 111(11) (2014) 2326-37.

[30] J.M. Heath, J. Fernandez Esmerats, L. Khambouneheuang, S. Kumar, R. Simmons, H. Jo, Mechanosensitive microRNA-181b Regulates Aortic Valve Endothelial Matrix Degradation by Targeting TIMP3, Cardiovasc Eng Technol 9(2) (2018) 141-150.

[31] J. Fernandez Esmerats, N. Villa-Roel, S. Kumar, L. Gu, M.T. Salim, M. Ohh, W.R. Taylor, R.M. Nerem, A.P. Yoganathan, H. Jo, Disturbed Flow Increases UBE2C (Ubiquitin E2 Ligase C) via Loss of miR-483-3p, Inducing Aortic Valve Calcification by the pVHL (von Hippel-Lindau Protein) and HIF-1alpha (Hypoxia-Inducible Factor-1alpha) Pathway in Endothelial Cells, Arterioscler Thromb Vasc Biol 39(3) (2019) 467-481.

[32] G. Zhong, S. Su, J. Li, H. Zhao, D. Hu, J. Chen, S. Li, Y. Lin, L. Wen, X. Lin, G. Xian, D. Xu, Q. Zeng, Activation of Piezo1 promotes osteogenic differentiation of aortic valve interstitial cell through YAP-dependent glutaminolysis, Sci Adv 9(22) (2023) eadg0478.

[33] K. Balachandran, S. Konduri, P. Sucosky, H. Jo, A.P. Yoganathan, An ex vivo study of the biological properties of porcine aortic valves in response to circumferential cyclic stretch, Ann Biomed Eng 34(11) (2006) 1655-65.

[34] W.D. Merryman, H.D. Lukoff, R.A. Long, G.C. Engelmayr, Jr., R.A. Hopkins, M.S. Sacks, Synergistic effects of cyclic tension and transforming growth factor-beta1 on the aortic valve myofibroblast, Cardiovasc Pathol 16(5) (2007) 268-76.

[35] K. Balachandran, P. Sucosky, H. Jo, A.P. Yoganathan, Elevated cyclic stretch alters matrix remodeling in aortic valve cusps: implications for degenerative aortic valve disease, Am J Physiol Heart Circ Physiol 296(3) (2009) H756-64.

[36] K.E. Smith, S.A. Metzler, J.N. Warnock, Cyclic strain inhibits acute pro-inflammatory gene expression in aortic valve interstitial cells, Biomech Model Mechanobiol 9(1) (2010) 117-25.

[37] K. Balachandran, P. Sucosky, H. Jo, A.P. Yoganathan, Elevated cyclic stretch induces aortic valve calcification in a bone morphogenic protein-dependent manner, Am J Pathol 177(1) (2010) 49-57.

[38] P. Thayer, K. Balachandran, S. Rathan, C.H. Yap, S. Arjunon, H. Jo, A.P. Yoganathan, The effects of combined cyclic stretch and pressure on the aortic valve interstitial cell phenotype, Ann Biomed Eng 39(6) (2011) 1654-67.

[39] K. Balachandran, M.A. Bakay, J.M. Connolly, X. Zhang, A.P. Yoganathan, R.J. Levy, Aortic valve cyclic stretch causes increased remodeling activity and enhanced serotonin receptor responsiveness, Ann Thorac Surg 92(1) (2011) 147-53.

[40] K. Balachandran, S. Hussain, C.H. Yap, M. Padala, A.H. Chester, A.P. Yoganathan, Elevated cyclic stretch and serotonin result in altered aortic valve remodeling via a mechanosensitive 5-HT(2A) receptor-dependent pathway, Cardiovasc Pathol 21(3) (2012) 206-13.

[41] J.D. Hutcheson, R. Venkataraman, F.J. Baudenbacher, W.D. Merryman, Intracellular Ca(2+) accumulation is strain-dependent and correlates with apoptosis in aortic valve fibroblasts, J Biomech 45(5) (2012) 888-94.

[42] R.A. Gould, K. Chin, T.P. Santisakultarm, A. Dropkin, J.M. Richards, C.B. Schaffer, J.T. Butcher, Cyclic strain anisotropy regulates valvular interstitial cell phenotype and tissue remodeling in three-dimensional culture, Acta Biomater 8(5) (2012) 1710-9.

[43] C.I. Fisher, J. Chen, W.D. Merryman, Calcific nodule morphogenesis by heart valve interstitial cells is strain dependent, Biomech Model Mechanobiol 12(1) (2013) 5-17.

[44] C. Moraes, M. Likhitpanichkul, C.J. Lam, B.M. Beca, Y. Sun, C.A. Simmons, Microdevice array-based identification of distinct mechanobiological response profiles in layer-specific valve interstitial cells, Integr Biol (Camb) 5(4) (2013) 673-80.

[45] Z. Ferdous, H. Jo, R.M. Nerem, Strain magnitude-dependent calcific marker expression in valvular and vascular cells, Cells Tissues Organs 197(5) (2013) 372-83.

[46] J. Chen, L.M. Ryzhova, M.K. Sewell-Loftin, C.B. Brown, S.S. Huppert, H.S. Baldwin, W.D. Merryman, Notch1 Mutation Leads to Valvular Calcification Through Enhanced Myofibroblast Mechanotransduction, Arterioscler Thromb Vasc Biol 35(7) (2015) 1597-605.

[47] N.T. Lam, T.J. Muldoon, K.P. Quinn, N. Rajaram, K. Balachandran, Valve interstitial cell contractile strength and metabolic state are dependent on its shape, Integr Biol (Camb) 8(10) (2016) 1079-1089.

[48] S.A. Metzler, S.C. Waller, J.N. Warnock, Quantitative Characterization of Aortic Valve Endothelial Cell Viability and Morphology In Situ Under Cyclic Stretch, Cardiovasc Eng Technol 10(1) (2019) 173-180.

[49] M.T. Salim, J.F. Esmerats, S. Arjunon, N. Villa-Roel, R.M. Nerem, H. Jo, A.P. Yoganathan, miR-214 is Stretch-Sensitive in Aortic Valve and Inhibits Aortic Valve Calcification, Ann Biomed Eng 47(4) (2019) 1106-1115.

[50] M.T. Salim, N. Villa-Roel, B. Vogel, H. Jo, A.P. Yoganathan, HIF1A inhibitor PX-478 reduces pathological stretch-induced calcification and collagen turnover in aortic valve, Front Cardiovasc Med 9 (2022) 1002067.

[51] T.M. West, D.P. Howsmon, M.W. Massidda, H.N. Vo, A.A. Janobas, A.B. Baker, M.S. Sacks, The effects of strain history on aortic valve interstitial cell activation in a 3D hydrogel environment, APL Bioeng 7(2) (2023) 026101.

[52] R. Zhao, K.L. Sider, C.A. Simmons, Measurement of layer-specific mechanical properties in multilayered biomaterials by micropipette aspiration, Acta Biomater 7(3) (2011) 1220-7.

[53] M.K. Sewell-Loftin, C.B. Brown, H.S. Baldwin, W.D. Merryman, A novel technique for quantifying mouse heart valve leaflet stiffness with atomic force microscopy, J Heart Valve Dis 21(4) (2012) 513-20.

[54] H. Wang, M.W. Tibbitt, S.J. Langer, L.A. Leinwand, K.S. Anseth, Hydrogels preserve native phenotypes of valvular fibroblasts through an elasticity-regulated PI3K/AKT pathway, Proc Natl Acad Sci U S A 110(48) (2013) 19336-41.

[55] H. Maleki, M.G. Doyle, M. Chehade, Q. Liu, C.A. Simmons, The focal mechanical properties of normal and diseased porcine aortic valve tissue measured by a novel microindentation device, J Mech Behav Biomed Mater 115 (2021) 104245.

[56] C.Y. Yip, J.H. Chen, R. Zhao, C.A. Simmons, Calcification by valve interstitial cells is regulated by the stiffness of the extracellular matrix, Arterioscler Thromb Vasc Biol 29(6) (2009) 936-42.

[57] A.M. Kloxin, J.A. Benton, K.S. Anseth, In situ elasticity modulation with dynamic substrates to direct cell phenotype, Biomaterials 31(1) (2010) 1-8.

[58] J.H. Chen, W.L. Chen, K.L. Sider, C.Y. Yip, C.A. Simmons, beta-catenin mediates mechanically regulated, transforming growth factor-beta1-induced myofibroblast differentiation of aortic valve interstitial cells, Arterioscler Thromb Vasc Biol 31(3) (2011) 590-7.

[59] C.M. Kirschner, D.L. Alge, S.T. Gould, K.S. Anseth, Clickable, photodegradable hydrogels to dynamically modulate valvular interstitial cell phenotype, Adv Healthc Mater 3(5) (2014) 649-57.

[60] S.T. Gould, E.E. Matherly, J.N. Smith, D.D. Heistad, K.S. Anseth, The role of valvular endothelial cell paracrine signaling and matrix elasticity on valvular interstitial cell activation, Biomaterials 35(11) (2014) 3596-606.

[61] L.R. Balaoing, A.D. Post, A.Y. Lin, H. Tseng, J.L. Moake, K.J. Grande-Allen, Laminin Peptide-Immobilized Hydrogels Modulate Valve Endothelial Cell Hemostatic Regulation, PLoS One 10(6) (2015) e0130749.

[62] H. Ma, A.R. Killaars, F.W. DelRio, C. Yang, K.S. Anseth, Myofibroblastic activation of valvular interstitial cells is modulated by spatial variations in matrix elasticity and its organization, Biomaterials 131 (2017) 131-144.

[63] S. Dahal, P. Huang, B.T. Murray, G.J. Mahler, Endothelial to mesenchymal transformation is induced by altered extracellular matrix in aortic valve endothelial cells, J Biomed Mater Res A 105(10) (2017) 2729-2741.

[64] A. Zhong, Z. Mirzaei, C.A. Simmons, The Roles of Matrix Stiffness and ss-Catenin Signaling in Endothelial-to-Mesenchymal Transition of Aortic Valve Endothelial Cells, Cardiovasc Eng Technol 9(2) (2018) 158-167.

[65] A. Khang, A. Gonzalez Rodriguez, M.E. Schroeder, J. Sansom, E. Lejeune, K.S. Anseth, M.S. Sacks, Quantifying heart valve interstitial cell contractile state using highly tunable poly(ethylene glycol) hydrogels, Acta Biomater 96 (2019) 354-367.

[66] C.J. Walker, C. Crocini, D. Ramirez, A.R. Killaars, J.C. Grim, B.A. Aguado, K. Clark, M.A. Allen, R.D. Dowell, L.A. Leinwand, K.S. Anseth, Nuclear mechanosensing drives chromatin remodelling in persistently activated fibroblasts, Nat Biomed Eng 5(12) (2021) 1485-1499.

[67] S. Dahal, J.A. Bramsen, B.R. Alber, B.T. Murray, P. Huang, M.H. Chen, G.J. Mahler, Chondroitin Sulfate Promotes Interstitial Cell Activation and Calcification in an In Vitro Model of the Aortic Valve, Cardiovasc Eng Technol 13(3) (2022) 481-494.

[68] D. Batan, D.K. Peters, M.E. Schroeder, B.A. Aguado, M.W. Young, R.M. Weiss, K.S. Anseth, Hydrogel cultures reveal Transient Receptor Potential Vanilloid 4 regulation of myofibroblast activation and proliferation in valvular interstitial cells, FASEB J 36(5) (2022) e22306.

[69] C.J. Walker, D. Batan, C.T. Bishop, D. Ramirez, B.A. Aguado, M.E. Schroeder, C. Crocini, J. Schwisow, K. Moulton, L. Macdougall, R.M. Weiss, M.A. Allen, R. Dowell, L.A. Leinwand, K.S. Anseth, Extracellular matrix stiffness controls cardiac valve myofibroblast activation through epigenetic remodeling, Bioeng Transl Med 7(3) (2022) e10394.
